# Glutamate indicators with improved activation kinetics and localization for imaging synaptic transmission

**DOI:** 10.1038/s41592-023-01863-6

**Published:** 2023-05-04

**Authors:** Abhi Aggarwal, Rui Liu, Yang Chen, Amelia J. Ralowicz, Samuel J. Bergerson, Filip Tomaska, Boaz Mohar, Timothy L. Hanson, Jeremy P. Hasseman, Daniel Reep, Getahun Tsegaye, Pantong Yao, Xiang Ji, Marinus Kloos, Deepika Walpita, Ronak Patel, Manuel A. Mohr, Paul W. Tillberg, The GENIE Project Team, Loren L. Looger, Jonathan S. Marvin, Michael B. Hoppa, Arthur Konnerth, David Kleinfeld, Eric R. Schreiter, Kaspar Podgorski

**Affiliations:** 1 Janelia Research Campus, Howard Hughes Medical Institute, Ashburn, VA USA; 2 Allen Institute for Neural Dynamics, Seattle, WA USA; 3 Department of Physics, University of California, San Diego, La Jolla, CA USA; 4 Institute of Neuroscience and Cluster for Systems Neurology (SyNergy), Technical University of Munich (TUM), Munich, Germany; 5 Department of Biological Sciences, Dartmouth College, Hanover, NH USA; 6 Department of Physiology, Second Faculty of Medicine, Charles University, Prague, Czech Republic; 7 Neurosciences Graduate Program, University of California San Diego, La Jolla, CA USA; 8 Department of Biosystems Science and Engineering, Swiss Federal Institute of Technology (ETH) Zurich, Basel, Switzerland; 9 Howard Hughes Medical Institute, Department of Neurosciences, University of California, San Diego, La Jolla, CA USA; 10 Section of Neurobiology, University of California, San Diego, La Jolla, CA USA

**Keywords:** Synaptic transmission, Fluorescent proteins, Mouse

## Abstract

The fluorescent glutamate indicator iGluSnFR enables imaging of neurotransmission with genetic and molecular specificity. However, existing iGluSnFR variants exhibit low in vivo signal-to-noise ratios, saturating activation kinetics and exclusion from postsynaptic densities. Using a multiassay screen in bacteria, soluble protein and cultured neurons, we generated variants with improved signal-to-noise ratios and kinetics. We developed surface display constructs that improve iGluSnFR’s nanoscopic localization to postsynapses. The resulting indicator iGluSnFR3 exhibits rapid nonsaturating activation kinetics and reports synaptic glutamate release with decreased saturation and increased specificity versus extrasynaptic signals in cultured neurons. Simultaneous imaging and electrophysiology at individual boutons in mouse visual cortex showed that iGluSnFR3 transients report single action potentials with high specificity. In vibrissal sensory cortex layer 4, we used iGluSnFR3 to characterize distinct patterns of touch-evoked feedforward input from thalamocortical boutons and both feedforward and recurrent input onto L4 cortical neuron dendritic spines.

## Abstract (web summary)

iGluSnFR variants with improved signal-to-noise ratios and targeting to postsynaptic sites have been developed, enabling the analysis of glutamatergic neurotransmission in vivo as illustrated in the mouse visual and somatosensory cortex.

## Main

Glutamate is the most abundant neurotransmitter in the vertebrate brain, with approximately one glutamatergic synapse per cubic micrometer of neuropil^1,2^. Despite such close packing, synaptic glutamate signaling incurs little crosstalk^3^. Synapses achieve this spatial specificity partly through the kinetics and localization of ionotropic glutamate receptors. Glutamate is released when vesicles fuse to the presynaptic membrane, producing brief, localized concentration transients that are sensed by closely apposed postsynaptic glutamate receptors with low affinity and rapid activation kinetics^4,5^. Synaptic glutamate is quickly taken up by glutamate transporters, but some escapes the synaptic cleft and signals extra-synaptically via high-affinity receptors^3^. Methods are needed to measure distinct signals at many synapses in vivo to study the nature of dendritic integration, including how inputs are arranged^6,7^, mechanisms governing synaptic plasticity^8,9^ and neuronal computation^10,11^. Such methods should have high sensitivity to monitor many synapses simultaneously deep in tissue over single trials, high spatial specificity to record from distinct synapses, low saturation to quantify release and fast kinetics.

The intensity-based Glutamate-Sensing Fluorescent Reporter (iGluSnFR) and its variants have been widely adopted to image synaptic activity, including spontaneous and evoked quantal release^12–16^, and extrasynaptic signaling^17,18^. However, existing iGluSnFR variants have low signal-to-noise ratio (SNR) in vivo, limiting how many synapses can be monitored simultaneously. Moreover, although experiments have bounded the spatial extent of iGluSnFR signals^10,13,18–21^, these bounds are large. For example, SF-Venus-iGluSnFR.A184S signal correlations span 3.6 μm in vivo^18^, a volume containing roughly 30 glutamatergic synapses^1,2^. It is therefore unclear, from iGluSnFR measurements alone, whether a signal originates from a synaptic partner or a nearby unconnected axon. Measurements and simulations suggest that this large spatial extent is caused by saturation of ON rates^20,22^ near release sites (Supplementary Note 1). The glutamate concentration that half-saturates ON rates, *K*_fast_, is distinct from *K*_D_, the concentration that half-saturates steady-state fluorescence. Experiments also suggested that iGluSnFR’s membrane-display construct (PDGFR) has poor nanoscopic localization to postsynapses; signals are larger on axons than dendrites^20^ and fusion to a postsynaptic protein increased signals of a low-affinity variant^13^.

We sought to engineer iGluSnFR variants with increased sensitivity and improved specificity for synaptic versus extrasynaptic glutamate signals. We performed 20 rounds of diversification and selection in bacteria (roughly 10^6^ variants screened) and lysate (roughly 10^4^ variants screened), with the final two rounds including assays in cultured neurons (roughly 10^3^ variants screened), while selecting for kinetic and photophysical properties predicted to improve synapse-specific signals. With a selected variant, iGluSnFR3.v857, we screened C-terminal membrane-display domains to identify variants with improved synaptic responses, then investigated their nanoscopic localization to postsynapses in vivo. We assessed saturation and synaptic specificity of iGluSnFR variants in neuronal culture by manipulating vesicle release and puffing glutamate onto neurons. In mouse cortex in vivo, we characterized responses to action potentials (APs) using simultaneous patch-clamp electrophysiology and imaging, and to awake whisker stimulation. Finally, we used iGluSnFR3 to compare spatiotemporal activity patterns of feedforward thalamocortical and recurrent inputs to layer 4 (L4), 400 μm below the pia. Thalamocortical boutons exhibit low-latency responses phase-locked to whisker stimulation, while L4 neuron spines in the same compartment each exhibit characteristic timings varying over tens of milliseconds, reflecting the timing distribution of recurrent inputs. The measurement of these distinct patterns on densely intertwined neuronal structures demonstrates the spatial specificity of iGluSnFR3 for synaptic versus extrasynaptic glutamate.

## Results

### iGluSnFR3 engineering

We started our screen with SF-Venus-iGluSnFR-A184V (hereafter, wild-type, WT), a yellow variant with large two-photon (2P) cross-section^20^ at wavelengths over 1,000 nm (ref. ^23^) and the highest *K*_fast_ among superfolder variants (Supplementary Note 1). We evolved indicators through 20 rounds of mutagenesis and screening in bacteria, purified protein and cultured neurons (Supplementary Note 2). We used high-throughput assays to discard unwanted variants followed by lower-throughput assays to richly characterize variants without compromising library size.

**Fig. 1:**
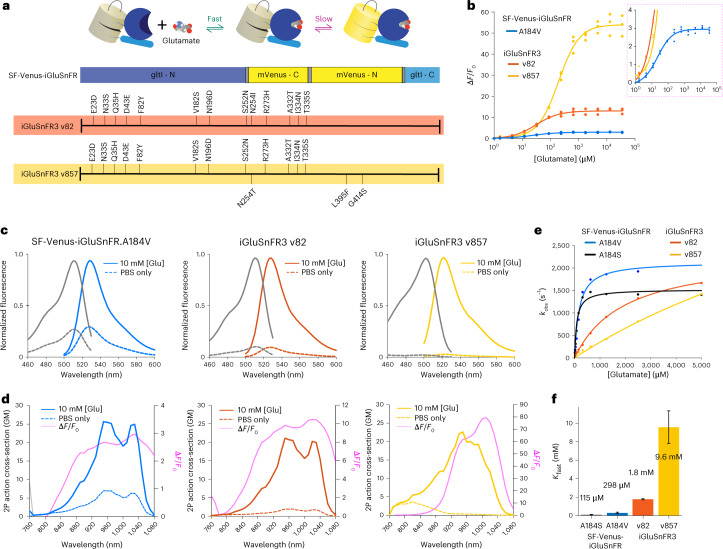
Photophysical properties of iGluSnFR3 variants. **a**, The top shows that iGluSnFR consists of a circularly permuted fluorescent protein (yellow) inserted between interacting N- and C-terminal domains of the bacterial periplasmic glutamate–aspartate binding protein gltI (blue). The dim unbound indicator rapidly binds glutamate, then undergoes a slower transition to a highly fluorescent state. The bottom shows the locations of amino acid substitutions of iGluSnFR3 variants v82 (red) and v857 (yellow) relative to the parent variant SF-Venus.iGluSnFR.A184V. **b**, Glutamate titrations of purified iGluSnFR protein. The right panel is a zoom-in view. *n* = 3 titration series. **c**, One-photon excitation and emission spectra of soluble SF-Venus-iGluSnFR.A184V, v82 and v857 in the presence (10 mM) and absence of glutamate. **d**, 2P excitation spectra and Δ*F*/*F*_0_ of the iGluSnFR3 variants in the presence (10 mM) and absence of glutamate. Pink lines are computed Δ*F*/*F*_0_. **e**, Stopped-flow measurements of initial ON rate (*k*_obs_) showing different degrees of kinetic saturation for SF-iGluSnFR and iGluSnFR3 variants. **f**, Estimated *K*_fast_, the glutamate concentration that half-saturates the indicator’s initial rapid binding equilibrium. The inferred value of *K*_fast_ for v857 is outside the tested concentration range, making it uncertain. Error bars denote least squares fit ± s.e.m.

**Fig. 2:**
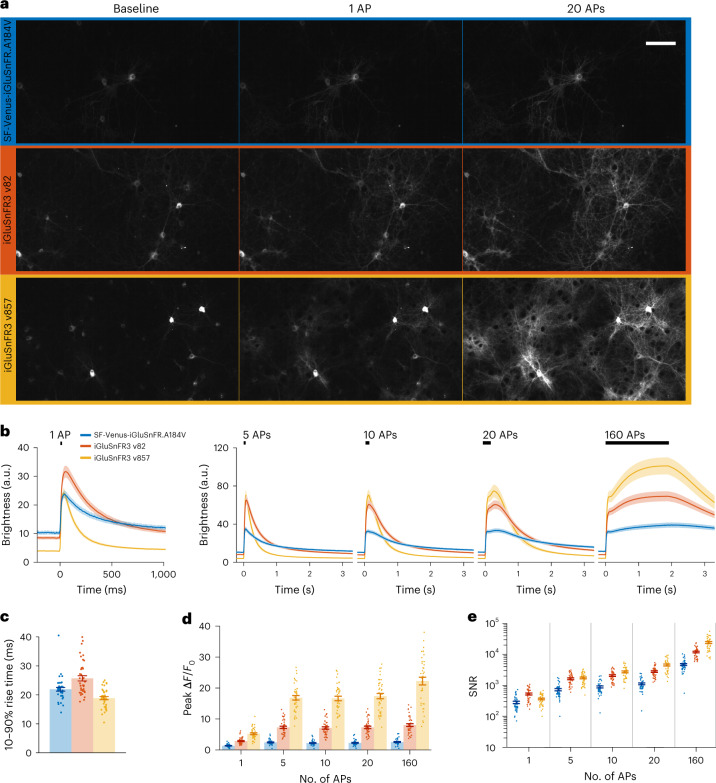
Characterization of iGluSnFR3 in cultured neurons. **a**, Images of primary cultures expressing surface-displayed SF-Venus-iGluSnFR-A184V (blue), v82 (red) and v857 (yellow) under hSyn promoter before stimulation and at peak brightness after field stimulations of 1 and 20 APs, at 21 °C. Scale bar, 100 μm. **b**, Mean pixel brightness traces for stimulations of 1, 5, 10, 20 and 160 APs at 80 Hz. **c**, 10–90% rise time of the three variants for the 1 AP condition. **d**, Peak Δ*F*/*F*_0_ of the three variants across conditions. **e**, Time-integrated SNR for the three variants across conditions. Traces and error bars denote mean ± s.e.m., *n* = 40 culture wells for each variant.

In each round, the input population was diversified by error-prone PCR, recombination or site-directed mutagenesis. We screened each library in bacterial colonies (roughly 10^5^ per round) under 2P (rounds 5–8) or one-photon (other rounds) excitation, selecting bright colonies to retain fast-maturing variants with high glutamate-bound brightness ([Glu]_cytosolic_ ≅ 100 mM in *Escherichia coli*). For these variants (roughly 400 per round) we measured fluorescence spectra in clarified lysate before and after adding glutamate, retaining only those with large responses and desirable spectral properties. For 4–20 variants per round, we measured (in various rounds): affinity, pH response, quantum yield, extinction coefficient, 2P spectra, fluorescence correlation spectroscopy (FCS) and stopped-flow kinetics. In the final two rounds, winners from several previous rounds were displayed on cultured rat neurons^24^, where we measured brightness, membrane trafficking, bleaching and responses to field-stimulated APs using high-speed (180 frames per s) imaging. We selected iGluSnFR3.v857 (‘v857’, 15 mutations versus WT; Supplementary Note 3) for its high *K*_fast_, rapid on-cell kinetics, brightness, fractional fluorescence response (Δ*F*/*F*_0_) and photostability (Figs. 1a–e and 2a–e). We selected the closely related variant iGluSnFR3.v82 (‘v82’, 13 mutations versus WT) for comparison, for its large one-AP response amplitude but kinetic properties less favorable for synaptic specificity.

**Extended Data Fig. 1:**
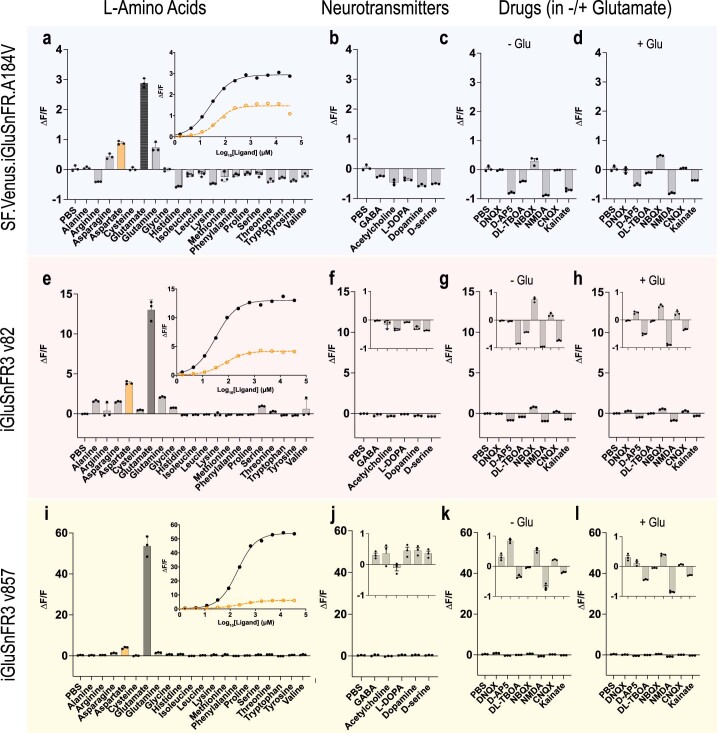
*In vitro* specificity of soluble proteins SF-Venus-iGluSnFR-A184V (blue, top), iGluSnFR3.v82 (red, middle) and iGluSnFR3.v857 (yellow, bottom). **a, e, i**) ∆F/F_0_ of the three biosensors for additions of L-amino acids (10 mM concentration, pH 7.3, buffered in PBS). Zoom-in shows titrations for glutamate (black) and aspartate (orange). **b, f, j**) ∆F/F_0_ for additions of common neurotransmitters (GABA, Acetylcholine, L-DOPA, Dopamine, D-serine; 10 mM concentration, pH 7.3, buffered in PBS). **c, g, k**) ∆F/F_0_ for additions of glutamatergic drugs (DNQX [10 mM], D-AP5 [500 µM], DL-TBOA [4 mM], NBQX [1 mM], NMDA [10 mM], CNQX [4 mM], Kainate [4 mM]) in the absence of glutamate and **d, h, l**) in presence of glutamate (10 mM, pH 7.3).

**Extended Data Fig. 2:**
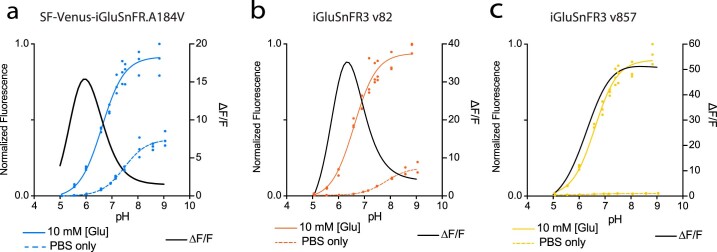
*In vitro* pH titration of soluble proteins. **a**) SF-Venus-iGluSnFR-A184V (left), **b**) iGluSnFR3 v82 (middle) and **c**) iGluSnFR v857 (right). Solid lines: saturating glutamate (10 mM, pH 7.3 buffered in PBS); Dotted lines: absence of glutamate. Sigmoidal fits are overlaid. Black lines show ∆F/F_0_ as a function of pH. All measurements were made using purified soluble protein; N = 3 titration series of a single protein sample for each measurement. Data obtained from fitting are presented in Supplementary Table 1.

**Extended Data Fig. 3:**
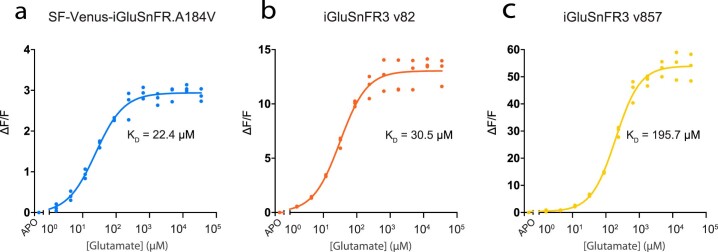
*In vitro* glutamate titration of soluble proteins. **a**) SF-Venus-iGluSnFR.A184V, **b**) v82 and **c**) v857 (pH 7.3), with corresponding fits and dissociation constants (K_D_). N = 3 titration series of a single protein sample for each variant.

**Extended Data Fig. 4:**
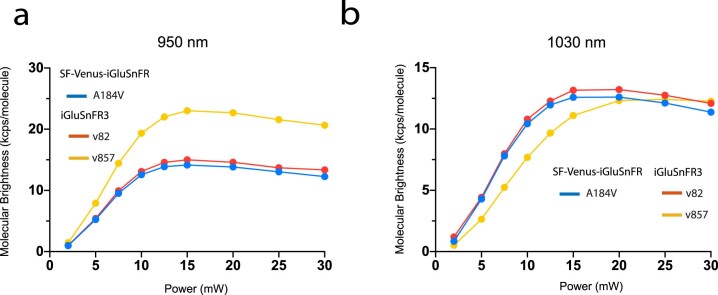
Molecular brightness (kcps/molecule) of iGluSnFR variants. Measurements using fluorescence correlation spectroscopy (FCS) at **a**) 950 nm and **b**) 1030 nm at varying power. All measurements were made using purified soluble protein in the glutamate-bound state.

As soluble proteins, v82 and v857 have less-saturating activation kinetics than WT, with estimated *K*_fast_ values 6 and 33 times larger, respectively (Supplementary Note 1 and Fig. 1d,e). Both show increased fluorescence response to saturating glutamate (Fig. 1b,c), due largely to dimmer glutamate-free states (Extended Data Table 1), and have high specificity for glutamate over other neurotransmitters, amino acids and drugs (Extended Data Fig. 1). v857 has an SYG rather than GYG chromophore sequence (Supplementary Note 3), a blue-shifted fluorescence spectrum, larger glutamate-bound quantum yield, lower extinction coefficient, reduced pH sensitivity of the unbound state, reduced affinity in vitro, larger 2P action cross-section and higher 2P-excited molecular brightness measured by FCS (Extended Data Table 1 and Extended Data Figs. 2–4). v857 exhibits strong quantum yield increase on glutamate binding, potentially useful for generating fluorescence lifetime indicators^25^.

**Extended Data Fig. 5:**
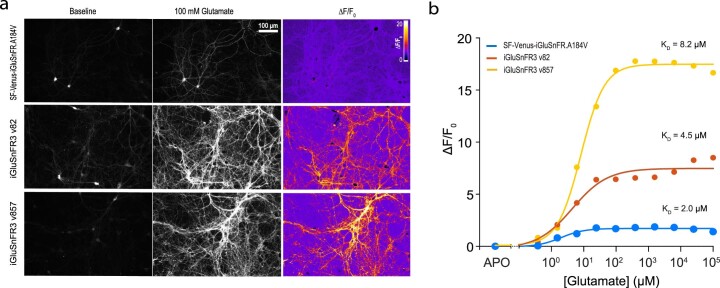
Glutamate titrations. **a**) Titrations of WT (SF-Venus-iGluSnFR-A184V), iGluSnFR3.v82 and iGluSnFR3.v857 on the surface of cultured hippocampal neurons. Baseline, 100 mM glutamate, and ∆F/F_0_ heat maps shown. **b**) ∆F/F_0_ affinity curves. ‘APO’ represents brightness in imaging buffer without added glutamate. ∆F/F_0_ is computed as fractional change in intensity of entire field of view (FOV) relative to corresponding APO image. The points are fit using a sigmoidal curve.

In cultured neurons, surface-displayed v82 and v857 traffic well (Fig. 2a), show larger responses to field-stimulated APs (Fig. 2b,d), larger dynamic range and lower glutamate affinity than WT (Extended Data Fig. 5). v82 has a slightly slower and v857 a slightly faster one-AP rise time than WT (Fig. 2c). v857 and v82 show higher time-integrated SNRs than WT in all conditions tested (Fig. 2e).

Together, these results identify v857 as a sensitive, selective iGluSnFR variant with rapid on-neuron kinetics and high *K*_fast_. v82 is a sensitive, selective variant with higher affinity, slower on-neuron kinetics, and lower *K*_fast_ than v857: properties that should make it more responsive to extrasynaptic glutamate.

### Imaging of synaptic signals in neuronal culture

**Fig. 3:**
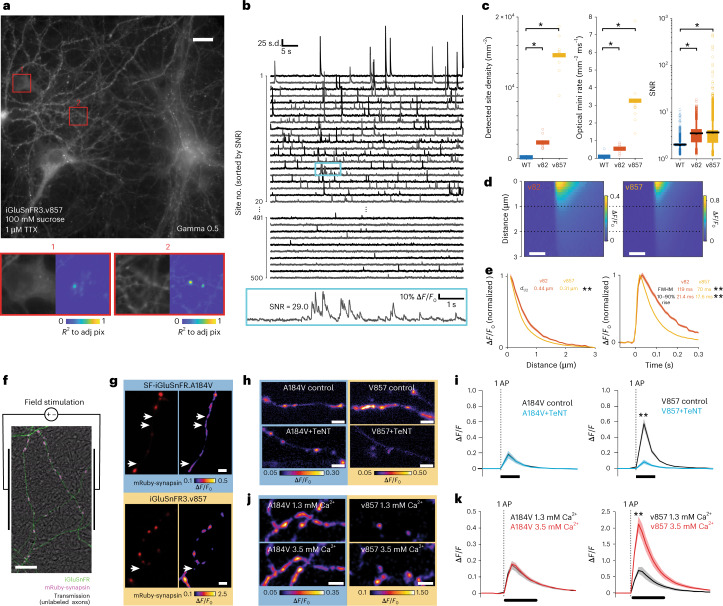
Imaging and manipulating synaptic release. **a**, The top shows a representative epifluorescence image of v857-expressing neuronal culture. Bottom, zoom of soma (1) and neurites (2) with corresponding correlation images showing release sites. **b**, Traces for individual sites in the same recording, each normalized to its standard deviation (s.d.), ordered by SNR. Sites ranked 1–20 and 491–500 are shown. The bottom shows a zoom of region highlighted in cyan above. **c**, Detected site density, event rate and SNR for WT and iGluSnFR3 variants expressed using hSyn.minDisplay. *n* = 9 FOV (v82 and v857); 8 FOV (WT), three culture wells per variant. Boxplots show mean (black line), 25th–75th percentile (box), 5th–95th percentile (whiskers), remaining points individual. *P < 0.01, two-sided Wilcoxon test versus WT. **d**, Kymographs of optical minis for v82 (left *n* = 96 sites) and v857 (right *n* = 2,862 sites). Scale bars, 100 ms. **e**, The left shows the optical mini Δ*F*/*F*_0_ as a function of distance from peak for v82 and v857, calculated one frame after onset. The right shows the optical mini Δ*F*/*F*_0_ as a function of time for v82 and v857, calculated one pixel from the center. *n* = 96 sites, v82; *n* = 2,862 sites, v857. Shading denotes s.e.m. *d*_1/2_ is the distance to half-maximum response. FWHM, full-width at half-maximum. **P < 1 × 10^−4^, bootstrap test (two-sided). **f**, Overlaid transmission (grayscale) and fluorescence images of sparsely transfected iGluSnFR+mRuby-synapsin culture. **g**, Representative mRuby-synapsin image (left) and iGluSnFR response (five APs at 50 Hz, right) for SF-iGluSnFR.A184V (top) and v857 (bottom). White arrows indicate putative crosstalk sites. **h**, Representative Δ*F*/*F*_0_ of SF-iGluSnFR.A184V and v857 for one-AP field stimulation at bath [Ca^2+^] of 1.3 and 3.5 mM. **i**, The one-AP (1AP) responses on cells expressing SF-iGluSnFR.A184V (left) or v857 (right) cotransfected with TeNT (cyan) or control (black). A184V: *n* = 16 cells, A184V + TeNT: *n* = 16 cells; v857: *n* = 16 cells; v857+TeNT: *n* = 16 cells. V857: *P* = 4.9 × 10^−4^; A184V: *P* = 0.83, two-sided paired Wilcoxon test. **j**, Representative Δ*F*/*F*_0_ images of SF-iGluSnFR.A184V and v857 in axons for 1AP field stimulation, control (top) and cotransfected with TeNT (bottom). **k**, 1AP evoked responses at individual boutons expressing SF-iGluSnFR.A184V (left) and v857 (right) with bath [Ca^2+^] of 1.3 mM (black) and 3.5 mM (red). *n* = 7 cells per variant. v857, *P* = 2.0 × 10^−3^; A184V, *P* = 0.13. Two-sided paired Wilcoxon test. **e**,**i**,**k**, Line denotes mean; shading denotes s.e.m. Scale bars, 20 μm (**a**); 8 μm (**f**); 100 ms (**i**,**k**); 2 μm (**g**,**h**,**j**).

**Extended Data Fig. 6:**
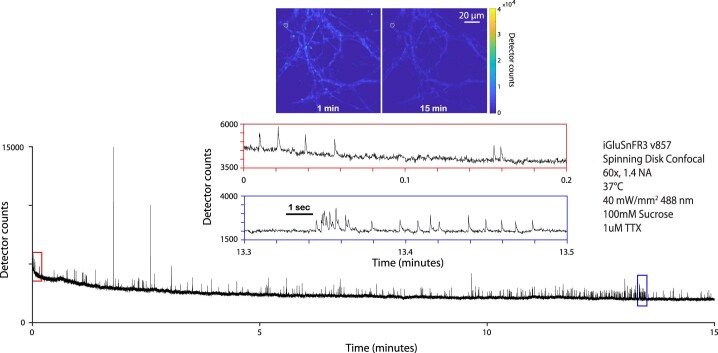
Long-duration recording of optical minis with iGluSnFR3.v857. Primary culture expressing iGluSnFR3.v857 was recorded with a spinning disk confocal microscope. (top) Inset images show frames from the beginning and end of the recording on the same intensity scale. Yellow circle denotes the site for which the traces below are plotted. Bottom, raw intensity trace for the highlighted site. Red and blue insets show zoom of highlighted regions at beginning and end of the recording, respectively.

We characterized sensitivity, spatial specificity and saturation by monitoring signals from spontaneous and evoked glutamate release at synapses between cultured neurons using widefield imaging. APs evoke synchronous release of varying numbers of vesicles per synapse. Additionally, spontaneous vesicle release produces asynchronous postsynaptic potentials (miniature release events, ‘minis’) typically corresponding to single vesicles^26,27^. With APs silenced (1 μM tetrodotoxin citrate (TTX)), synapses expressing v857 show large-amplitude asynchronous fluorescence transients (‘optical minis’^14,15^) (Supplementary Video 1 and Fig. 3a–c). As with electrophysiologically observed minis^27^, optical mini rates increase in hyperosmotic sucrose buffer (Supplementary Fig. 1). The high photostability of v857 allowed continuous 15-minute recordings of optical minis without reduction in SNR (Extended Data Fig. 6). v82 detected fewer optical minis than v857, while WT detected far fewer still (Fig. 3d). Optical minis of v857 had a narrower spatial extent than those of v82 (Fig. 3d,e; minis of WT could not be characterized due to low SNR), indicating that v82 undergoes kinetic saturation during minis (Supplementary Note 1).

To further assess spatial specificity, we recorded single-AP responses in dense cultures where sparse neurons coexpressed iGluSnFR and a release site marker (Ruby-synapsin) (Fig. 3f). Using SF-iGluSnFR.A184V, numerous sites of functional signals lacked mRuby labeling, indicating crosstalk from nearby unlabeled axons, which was greatly reduced using v857 (Fig. 3g). To quantitatively assess crosstalk, we compared responses when expressing either iGluSnFR alone or both iGluSnFR and tetanus toxin light chain (TeNT). TeNT cleaves synaptobrevin, blocking vesicle fusion^28^ (Supplementary Fig. 3). Axons coexpressing TeNT retained responses, likely corresponding to crosstalk. Amplitudes of crosstalk responses were greatly reduced in v857 + TeNT neurons (Fig. 3h,i) compared to v857 alone. In SF-iGluSnFR.A184V + TeNT coexpressing neurons, amplitudes were no smaller than SF-iGluSnFR.A184V alone.

To further assess saturation, we manipulated glutamate concentrations released onto the membrane. Release probability, and thus the average number of vesicles released, depends nonlinearly on extracellular [Ca^2+^] (refs. ^29,30^). Using v857, increasing extracellular [Ca^2+^] from 1.3 to 3.5 mM increased single-bouton one-AP Δ*F*/*F*_0_ responses from 70 ± 9 to 213 ± 24% (mean ± s.e.m., Fig. 3j,k), consistent with other measurements^29,30^. Under the same conditions, SF-iGluSnFR.A184V exhibited no significant increase in single-bouton response (*P* > 0.5, paired Wilcoxon), showing that it can saturate during synchronous release (Fig. 3j,k and Supplementary Fig. 3). We directly measured concentration-dependent on-neuron activation kinetics using puffs of glutamate from a pipette, finding that ON rates of v857 increase with pipette [glutamate] of 3–300 μM, whereas those of WT do not (Supplementary Fig. 2).

These results show that v857 more linearly reports glutamate release at individual synapses and better distinguishes synaptic from extrasynaptic signals in culture, whereas SF-iGluSnFR.A184V undergoes saturation that limits it in these respects.

### Improved membrane-display constructs

The spatial specificity of glutamate transmission relies on nanoscopic localization of receptors to postsynaptic densities apposed to presynaptic active zones. However, electrophysiology and imaging studies suggested iGluSnFR may be excluded from postsynaptic densities^13^. We reasoned that display constructs with improved localization would show improved synaptic responses. We compared display sequences while assessing the amplitude, SNR and detection rate of optical minis. We did not try to localize iGluSnFR exclusively to postsynapses, which requires low expression levels, risking susceptibility to bleaching.

**Fig. 4:**
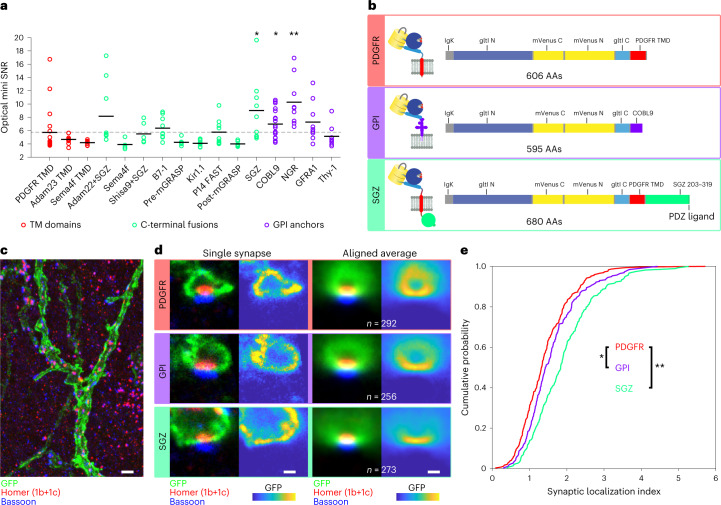
Membrane targeting sequence effects on synaptic responses and localization. **a**, SNR of optical minis in culture for v857, expressed using PDGFR transmembrane domain (pMinDisplay) and each of 15 other display constructs. Black lines denote means. *n* = 9 FOV over three wells per construct. *, **GPI_COBL9_, *P* = 0.0222; GPI_NGR_, *P* = 0.0003; SGZ, *P* = 0.0101; two-sided two-sample *t*-tests versus PDGFR. **b**, Sequence schematics for PDGFR, GPI and SGZ display constructs. PDGFR is a C-terminal fusion to the PDGFR transmembrane domain in the mammalian expression pMinDisplay vector. GPI contains a C-terminal glycosylphosphatidylinositol anchor. SGZ contains the PDGFR transmembrane domain, followed by a modified form of the cytosolic C-terminal domain of SGZ including a terminal PDZ ligand. **c**, Representative maximum intensity projection image of a portion of an expanded gel of mouse cortical tissue expressing v857.SGZ, immunostained for GFP (green), Homer1 (red) and Bassoon (blue). Scale bar, 5 μm expanded. **d**, Images of single representative synapses and aligned averages for the three iGluSnFR variants. For each image, immunostains for GFP (green), Homer1 (red) and Bassoon (blue) are shown on the left, and the GFP channel is shown on the right. Scale bars, 1 μm expanded. **e**, Cumulative probability plot of the ratio of GFP signal at the synapse center to that within a 3 × 6 µm^2^ (expanded) postsynaptic region. Means: PDGFR: 1.43 ± 0.05; GPI: 1.57 ± 0.05, *P* = 0.037; SGZ: 1.78 ± 0.04, *n* = 292 (PDGFR), 256 (GPI) and 273 (SGZ) synapses. *P < 0.05, **P < 0.01 Kolmogorov–Smirnov test (two-sided).

SnFR indicators are targeted to the outer membrane via insertion between an N-terminal IgΚ secretion leader sequence and C-terminal PDGFR transmembrane domain^19^. We compared v857 in this construct to 15 C-terminal variants including GPI anchors, transmembrane domains, cytosolic motifs and endoplasmic reticulum and Golgi export signals (Supplementary Table 1). Of these, two GPI anchors (GPI_COBL9_, GPI_NGR_) and a modified cytosolic fragment of Stargazin (SGZ) increased SNR of optical minis (Fig. 4a,b). These variants exhibited narrower optical minis and improved surface trafficking as measured by Δ*F*/*F* on glutamate application (Supplementary Fig. 4). A followup screen combining GPI_COBL9_ (henceforth, GPI) with 14 N-terminal leader sequences^31^ resulted in no further improvement compared to the existing IgK leader (Supplementary Fig. 4).

We next evaluated nanoscopic localization of these functionally improved constructs. We injected adeno-associated virus (AAV) encoding Cre-dependent SGZ, GPI and PDGFR variants of v857 into the cortex of *Emx1*-Cre mice. We performed expansion microscopy^32^ with immunolabeling for green fluorescent protein (GFP) (iGluSnFR), Bassoon (presynapse) and Homer1 (postsynapse) (Fig. 4c). For all variants, anti-GFP labeling was heterogeneously distributed within the membrane. However, reduced labeling of postsynapses specifically in PDGFR-labeled neurons was clearly observed in aligned average images over all identified synapses (Fig. 4d). Postsynaptic localization, that is, the ratio of anti-GFP signal at the synapse center to that within a 3 × 6 µm (expanded) postsynaptic region, was increased for GPI and SGZ constructs (Fig. 4e).

These results identify motifs that increase the SNR of synaptic signals, improve trafficking in cultured neurons, and improve iGluSnFR’s postsynaptic localization in vivo.

### In vivo iGluSnFR3 imaging and electrophysiology

**Extended Data Fig. 7:**
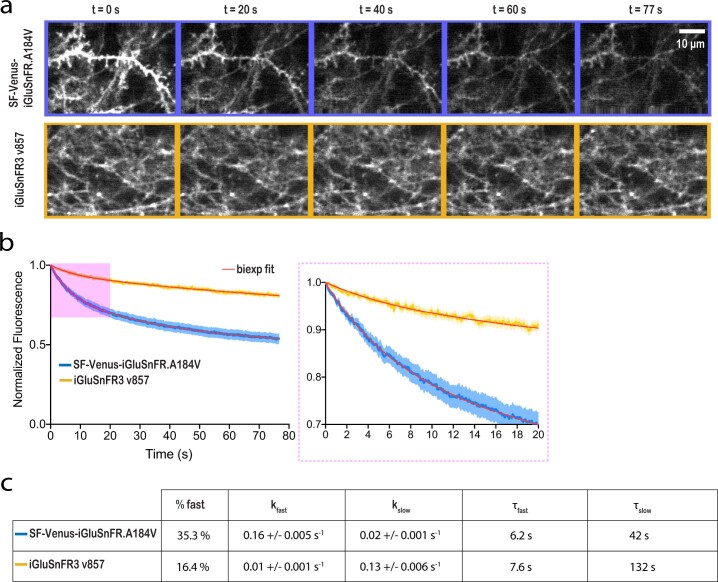
Photostability of iGluSnFR3 and SF-Venus-iGluSnFR.A184V under 2P excitation in vivo. **a**) Representative images of SF-Venus-iGluSnFR.A184V (blue; top) and iGluSnFR3.v857 (yellow; bottom), both in the PDGFR backbone, expressed in excitatory cortical neurons in mouse visual cortex, over the course of two-photon recordings at matched excitation power. Images are shown with the same brightness scale. **b**) Brightness, normalized to its initial value, of the two variants. Shading denotes s.e.m. A184V, n = 34 FOV’s from 5 mice; v857, n = 27 FOV’s from 5 mice. Curves are fit with two-phase exponential decay (red line). Right panel is a zoom-in view from 0 sec to 20 sec. **c**) Two-phase exponential decay parameters (+/−95% CI) of iGluSnFR3 and SF-Venus-iGluSnFR.A184V.

We next characterized iGluSnFR3 signals in mouse visual cortex. We observed strong signals in mice injected with AAVs encoding v857.GPI or v857.PDGFR variants, including 1-photon widefield recordings during visual stimulation (Supplementary Fig. 5), 2P recordings of spines and boutons (Supplementary Video 2), and increased photostability relative to WT (Extended Data Fig. 7). Although immunolabeling of virally expressed SGZ indicated strong expression and good membrane localization in mouse cortex (Fig. 4c,d), and bright expression and functional signals in rat cortical culture, SGZ exhibited dim native fluorescence and weak functional responses in mouse cortex (Supplementary Fig. 4). We therefore performed all remaining in vivo experiments with the PDGFR and GPI variants.

**Fig. 5:**
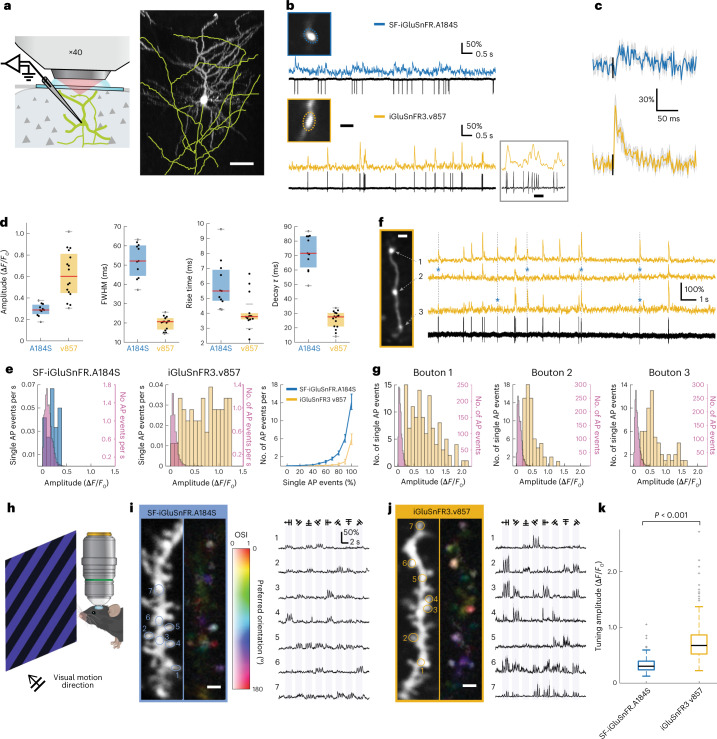
In vivo imaging and electrophysiology in visual cortex. **a**, The left shows the experimental schematic for simultaneous 2P axonal imaging and cell-attached recording in vivo. The right shows a *z*-projection of iGluSnFR3.v857-labeled neuron in mouse V1, with axon traced in yellow. Scale bar, 50 μm. **b**, 2P imaging of individual boutons and simultaneous cell-attached recordings from representative neurons labeled with SF-iGluSnFR.A184S (top, ‘A184S’) and v857 (bottom). The inset shows v857 response during a rapid spike train. Scale bars, 2 μm and 100 ms (inset). **c**, Spike-triggered averages for isolated APs measured with v857 (bottom) and A184S (top); mean ± s.e.m. of *n* = 10 consecutive events from recording in **b**. **d**, Amplitude (*P* = 1.1 × 10^−4^), full-width half-maximum (FWHM) (*P* = 1.6 × 10^−5^), rise time (*P* = 0.002) and decay *τ* (*P* = 1.6 × 10^−5^) of responses for isolated APs for v857 (*n* = 16 boutons from five neurons) and A184S (*n* = 11 boutons from five neurons); Wilcoxon tests (two-sided). **e**, The left and middle show histograms of amplitudes for single-AP events and in absence of APs for example boutons shown in **b**. The right shows a comparison of the non-AP event rate for v857 (yellow) and A184S (blue) as a function of detection threshold (mean ± s.e.m., *n* = 6 boutons for v857 and *n* = 5 boutons for A184S). **f**, Simultaneous v857 imaging (yellow) and cell-attached recording for three neighboring boutons recorded at 500 Hz each. Asterisks indicate putative synaptic release failures. Scale bar, 2 μm. **g**, Amplitude distribution of deconvolved AP responses for boutons shown in **f**. **h**, Experimental schematic for in vivo imaging of responses to visual motion stimuli in V1 dendrites. **i**, The left shows a L2/3 neuron dendrite expressing A184S and corresponding pixelwise tuning map (hue, preferred direction; saturation, OSI and lightness, response magnitude). The right shows the spine responses evoked by drifting gratings with directions indicated above. Numbers correspond to spines indicated at left. **j**, Same as **i** but from a v857-expressing neuron in a different mouse. Color scales for tuning map are identical to **i**. **k**, Response amplitudes for v857 and A184S (0.6140 (0.4620–0.8162) versus 0.2944 (0.2164–0.3925), *P* = 1.2 × 10^−67^, Wilcoxon test (two-sided), *n* = 261 ROI from five neurons for v857 and *n* = 375 ROI from five neurons for A184S). Boxplots denote median and interquartile range, outliers beyond 1.5 interquartile plotted separately. Scale bars in **i**,**j**, 2 μm.

Glutamate imaging is a promising approach for studying synaptic activity in the intact brain^10,18,20,33^ but the relationship between in vivo iGluSnFR signals and synaptic activity, such as presynaptic APs, remains incompletely characterized. We therefore imaged individual boutons on axons of individual sparsely labeled glutamatergic neurons during simultaneous cell-attached electrophysiological recordings from the parent cell bodies. Layer 2/3 pyramidal neurons in mouse primary visual cortex were electroporated with plasmids encoding either v857.GPI or SF-iGluSnFR.A184S. Cell-attached recordings and concurrent 2P imaging at 500 Hz frame rate were performed in lightly anesthetized, head-fixed mice (Fig. 5a,b). Boutons of v857-expressing neurons exhibited larger and faster one-AP-associated transients than those expressing SF-iGluSnFR.A184S (Fig. 5c,d). To quantify synaptic specificity, we compared transient amplitudes associated with single electrophysiologically observed APs to those not associated with APs. v857 showed a greater difference in these amplitudes than A184S (Fig. 5e). Using an amplitude threshold on denoised traces, 70% of presynaptic APs were detected with v857 at a false positive rate of 0.2 Hz, while 70% of presynaptic APs were detected with SF-iGluSnFR.A184S at a false positive rate of 3.7 Hz (Fig. 5f). At this threshold, we attribute most APs undetected by v857 to failures of synaptic release, since measurement noise was far lower than the variability in single-AP event amplitudes. We measured amplitude distributions of AP-associated fluorescence transients collected from each of three adjacent boutons during 1.5 min of continuous optical and electrophysiological recording (Fig. 5f,g). We next recorded from dendrites while presenting visual motion stimuli to lightly anesthetized mice (Fig. 5h). v857 reported direction-tuned responses more localized to dendritic spines and of larger amplitude than those reported by SF-iGluSnFR.A184S (Fig. 5i–k).

### Imaging ascending and recurrent inputs to barrel cortex L4

We next used iGluSnFR3 to study feedforward and recurrent inputs to vS1, the primary vibrissa (whisker) sensory cortex. Rodents rhythmically sweep their vibrissae to sense objects through touch^34^. Signals ascend from vibrissal follicles through ventral posteromedial (VPM) thalamus, then to L4 of vS1, where each vibrissa is represented by a column of neurons forming a characteristic ‘barrel’ structure. L4 excitatory cells within a barrel receive about 10% of their excitatory input from thalamocortical projections^35^, with most remaining (recurrent) connections originating from other L4 excitatory cells in the barrel^36,37^. The depth of L4 (roughly 350–450 µm) and the small synapse volume (<1 femtoliter) are challenges for measuring precisely timed synaptic activity, which we overcame using iGluSnFR3’s high SNR and direct wavefront sensing adaptive optics 2P (AO2P) microscopy^33^.

**Fig. 6:**
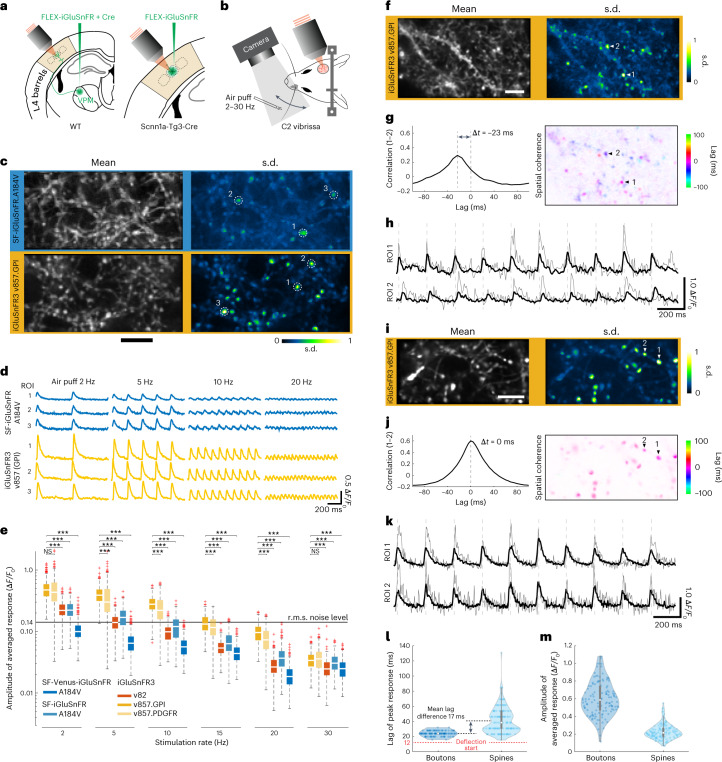
AO glutamate imaging of thalamocortical boutons and dendritic spines in vS1 L4. **a**, Schematic of AAV injection in VPM (left) in WT mice to express iGluSnFR on thalamocortical boutons and, separately, injection into vibrissa cortex (right) in *Scnn1a*-Tg3-Cre mice to express iGluSnFR on dendrites of L4 cortical neurons. **b**, Schematic of the air-puff stimulation in awake mice during high-speed AO2P imaging. Frame rates were roughly 250 Hz for boutons (**c**–**e**, **i**–**k**) and roughly 130 Hz for spines (**f**–**h**). **c**–**e**, Comparison of iGluSnFR variants in thalamocortical boutons across stimulation frequencies. **c**, The left shows an average image of thalamocortical axons in L4 labeled by SF-iGluSnFR.A184V (top) and v857.GPI (bottom). The right shows a normalized pixelwise standard deviation (s.d.) across 1-s averaged epochs from 60 trials. Scale bar, 10 μm. **d**, Mean responses (60 trials) for boutons shown in **c** labeled by v857.GPI (top) and A184V (bottom). **e**, Response amplitudes of boutons labeled with iGluSnFR3 and SF-iGluSnFR variants: v857.GPI (three mice, 389 boutons), v857.PDGFR (three mice, 314 boutons), v82.GPI (two mice, 312 boutons), A184V (three mice, 239 boutons) and Venus-A184V (two mice, 355 boutons). Line indicates root-mean-square (r.m.s.) noise level for v857.GPI. ***P < 0.001; NS, not significant; one-way analysis of variance followed by Bonferroni’s test. **f**–**h**, Measurement of response lags for v857.GPI-labeled L4 spines to 5 Hz vibrissal stimulation. **f**, Mean (left) and s.d. (right) images of L4 v857.GPI-labeled spines. Scale bar, 10 μm. **g**, The left shows a cross-correlation of signals from two active spines highlighted at right. The right shows a pixelwise lag of peak response relative to the recording’s dominant mode. **h**, Time series from spines identified in **g** with a representative trial in gray and mean in black. **i**–**k**, Same as **f**–**h** for thalamocortical boutons labeled with v857.GPI: mean and s.d. images scale bar, 10 μm (**i**), correlation and spatial coherence (**j**) and time series (**k**). **I**, Lag of peak response for thalamocortical boutons (123 ROI) and dendritic spines (145 ROI) in L4 vibrissal cortex, violin plot. A time of 0 denotes an electronic trigger to the air-puff valve, the red dashed line indicates the measured onset of vibrissal deflection. **m**, Response amplitudes of thalamocortical boutons (123 ROI) and dendritic spines (145 ROI), violin plot. (**e**,**l**,**m**) Boxplots denote median and interquartile range, outliers beyond 1.5 interquartiles are plotted separately.

**Extended Data Fig. 8:**
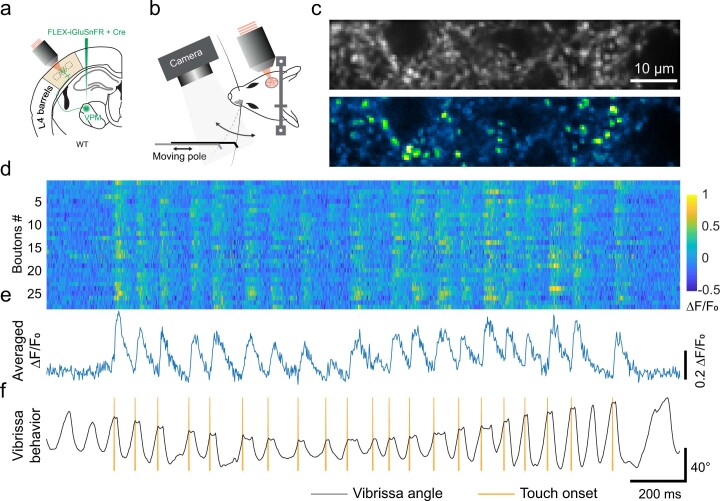
Functional *in vivo* imaging of layer 4 TC boutons during a pole touching task with iGluSnFR3.v857.GPI. **a**) Schematic of viral injection of AAV2/1.hSyn.iGluSnFR3.v857.GPI in VPM to label the TC boutons in layer 4. **b**) Schematic of the dynamic pole touch tasking during active sensing. The pole moves back and forth at a range of 5 mm along the azimuthal direction with an average speed of 1.25 mm/s. Images are acquired at the frame rate of ~500 Hz. **c**) Average (top) and s.d. (bottom) of the image of TC axons and boutons labeled by v857.GPI in layer 4 of vibrissa cortex. **d**) Raster plot of the touch response from individual boutons detected from panel c. **e**) Averaged touch response across all boutons in panel d. **f**) Corresponding whisking trace and touch onset. This experiment was performed with a single subject and is a representative session.

**Extended Data Fig. 9:**
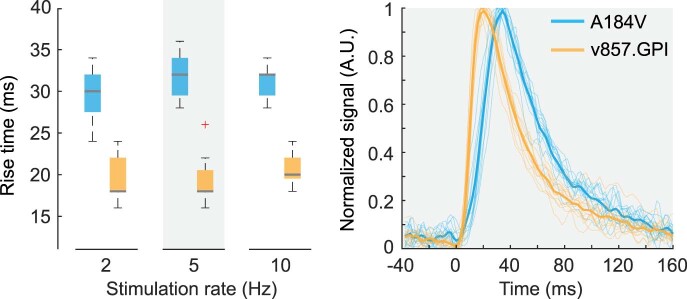
Rise time of SF-iGluSnFR.A184V and v857.GPI indicators for data shown in Fig. 6 c-d at stimulation rates of 2, 5 and 10 Hz. We extract the averaged response signal across multiple stimulations from 13 ROIs (including the ROIs 1 to 3 shown in Fig. 6c) in each dataset. Rise time (onset to peak) is manually detected from the averaged signal for each ROI. Left, rise time of the 13 ROIs in each dataset. Boxplot denotes median [interquartile range], with outliers beyond 1.5 IQR plotted individually. The plot on the right shows the normalized response to 5 Hz stimulation of each ROI (thin) and their average (thick) for SF-iGluSnFR.A184V and v857.GPI.

We expressed iGluSnFR variants in thalamocortical axons by injecting AAV into VPM (Fig. 6a). Thalamic boutons in L4 of awake, head-fixed mice showed large-amplitude transients induced by vibrissal touch during active whisking (Extended Data Fig. 8). To quantify thalamocortical responses across frequencies, we used air-puff stimulation (Fig. 6b) in awake mice (Fig. 6c and Supplementary Fig. 6 and Extended Data Fig. 9). Rhythmic stimulation from 2–30 Hz produced time-locked rapid-onset responses (Supplementary Video 3) that were characterized for five iGluSnFR variants across stimulation frequencies (Fig. 6d,e; 1,609 individual boutons, 13 mice). v857 showed greater modulation than SF-iGluSnFR variants at all frequencies, over twofold greater than SF.iGluSnFR.A184V and fivefold greater than WT for 2–20 Hz stimulation rates. Using v857, our AO2P microscope sampling at 125 Hz resolved single-trial responses at rates ≤15 Hz with SNR above 1.

We expressed v857.GPI in L4 dendrites by injecting Cre-dependent AAV into vS1 of *Scnn1a*-Cre mice^38^. We recorded spine responses (Fig. 6f), which should predominantly reflect recurrent input, while stimulating vibrissae at 5 Hz (Supplementary Video 4). Spine responses each showed characteristic lags differing across synapse pairs by up to 70 ms (ref. ^39^) (Fig. 6g,h). Similar comparisons across 123 thalamocortical boutons from two mice showed no time lag differences (Fig. 6i–k). As a population (145 dendritic spines from two mice), the lags of recurrent connections are broadly distributed with an average delay within L4 of 17 ms (Fig. 6l). The absence of heterogeneous time lags for the dense network of axons imaged in VPM-labeled mice further demonstrates the high specificity of v857 for synaptic versus extrasynaptic signals in vivo.

## Discussion

Glutamate imaging has long promised to complement dendritic Ca^2+^ imaging for recording spatially resolved synaptic inputs. Dendritic Ca^2+^ imaging reports postsynaptic signals governed largely by (nonlinear) *N*-methyl-d-aspartate (NMDA) receptor activation at spines^40^, but which are less-studied at nonspiny synapses and spatially mixed by propagating potentials and dendritic spikes^7^. Glutamate imaging avoids these confounds for studying spatial arrangements of inputs and their correlations, but imaging membrane signals demands especially sensitive and photostable indicators^41^, and exhibits confounds of synaptic crosstalk and kinetic saturation. iGluSnFR3 was engineered to improve all these properties.

Several limitations remain for iGluSnFR. As with previous generations, color variants are needed for multiplexing and affinity variants for different cells or compartments^20^. For measuring absolute analyte concentrations, fluorescence lifetime is more suitable than intensity contrast^25^. Achieving high spatial specificity with noncooperative indicators such as iGluSnFR requires high imaging speeds (Supplementary Note 1), whereas cooperative indicators could achieve this at lower frame rates. Although faster than WT, iGluSnFR3.v857 on-cell kinetics are slower than synaptic glutamate dynamics, and high-sensitivity variants of faster indicators (for example, iGlu_u_ (ref. ^22^), SF-iGluSnFR.S72A (ref. ^20^) are needed. The SGZ and GPI sequences reduce postsynaptic exclusion of iGluSnFR and improve spatial specificity but the SGZ construct performed poorly in vivo. Further evaluation of display sequences may yield yet better localization and function in vivo. Work is also needed to evaluate glutamate buffering effects on endogenous signaling^42^.

iGluSnFR3 provides advantages over previous methods. Its low kinetic saturation enables more quantitative measurements previously confounded by saturation effects. Its reliable detection of single electrophysiologically identified APs in vivo improves interpretation of signals and may facilitate in vivo studies of release parameters. Its SNR, kinetics and spatial specificity allow recording from populations of synapses while resolving milliseconds-differences in the timing of individual inputs. These capabilities will allow observation of synaptic dynamics underlying theories of neuronal computation and learning^9,11,43^.

## Methods

### Animal care and use statement

All experimental procedures involving animals were performed in accordance with protocols approved by the Institutional Animal Care and Use Committee at the respective institute ((HHMI Janelia Research Campus, Dartmouth College, University of California San Diego and TUM). Procedures in the United States conform to the National Institutes of Health (NIH) Guide for the Care and Use of Laboratory Animals. Procedures at TUM were approved by the state government of Bavaria, Germany. Mice were housed under controlled temperature (roughly 21 °C) and humidity (roughly 50%) conditions under a reverse light cycle.

HHMI Janelia Research Campus: protocols 14–115, 16–225

Dartmouth College: protocol 00002115

University of California, San Diego: protocol S02174M

Technical University of Munich: ROB-55.2-2532.Vet_02-17-181

This research has complied with all applicable ethical regulations.

### Molecular biology

Site-directed mutagenesis was performed using QuikChange (Agilent Technologies) or Q5-site-directed mutagenesis kit (New England Biolabs) according to the manufacturer’s suggestions. Error-prone PCR was done using Mutazyme PCR from Genemorph II mutagenesis kit (Agilent Technologies) according to the manufacturer’s suggestions, generating mean mutation rates of 4.5 to 9 DNA substitutions and/or clones. Recombination libraries were made using Staggered Extension PCR^44^. In each case, mutagenesis was performed on the coding sequence and reinserted into the same plasmid vector via Gibson assembly or digestion-ligation. Primers (Integrated DNA Technologies) used for PCR amplification of the iGluSnFR coding sequence were GATAAGGATCGATGGGGATCCGCCGCAGGCAGC and CCGGATCAAGCTTCGAATTCTTATTTCAGTGCCTT. Plasmid sequences were confirmed via Sanger sequencing.

### Construction of mammalian expression vectors

To express sensors on the cell surface, iGluSnFR coding sequences were PCR amplified from the bacterial expression (pRSET) constructs, followed by Gibson assembly into pAAV.hSyn (or Cre-dependent pAAV.hSyn.FLEX) vectors, into which the N- and C-terminal flanking sequences (IgK leader and surface display sequence) were first inserted. For the development of the display constructs library, the N-terminal leader or C-terminal surface display sequence was substituted using Gibson assembly. Recombinant AAV particles (rAAVs) were prepared by Viral Tools at Janelia according to their standard protocol. A list of iGluSnFR variants used in each experiment is presented in Supplementary Table 2.

### Construction of display construct library

Synthetic DNA consisting of different N-terminal secretion peptides and C-terminal anchoring domains were codon-optimized for *Mus musculus* and ordered as gene blocks from Integrated DNA Technologies (IDT). The fragments were PCR amplified and inserted into a pAAV.hSyn iGluSnFR vector via Gibson assembly. For the insertion of N-terminal secretion peptides, the vector backbone was created by digesting the pAAV.hSyn.iGluSnFR gene with *NheI* and *BamH1* to cut out IgK sequence and substituting the new fragments. For C-terminal anchors, the iGluSnFR gene was digested with *PstI* and *HindIII*, to cut out PDGFR and substituting the other anchors. The C-terminal libraries were cloned in combination with IgK as N-terminal secretion peptide, and N-terminal secretion peptide libraries were cloned with GPI_COBL9_ as the C-terminal anchoring domain. The C-terminal domains were selected from among sequences previously shown to improve secretion and membrane trafficking, and transmembrane domains from single-pass transmembrane proteins present in postsynaptic densities. Sequences and references are presented in Supplementary Table 1. The SGZ construct identified in the screen consists of the PDGFR transmembrane domain, followed by a short polybasic sequence (8 aa; MLWQKKPR), followed by residues 203–269 of mouse SGZ (UniProt no. Q71RJ2 and RefSeq no. NP_445803.1) previously identified as an intrinsic membrane-sorting signal^45^, and a six-residue PDZ ligand (6 aa; PQTTSV). We mutated 12 serine residues to aspartate (S221D, S228D, S237D, S239D, S240D, S241D, S243D, S247D, S249D, S253D, S265D and S269D (numbering with respect to mouse SGZ sequence)) to reduce the potential for phosphorylation.

### Bacterial colony and lysate measurements

iGluSnFR variants in soluble form in pRSET vector (Thermo Fisher Scientific) were transformed via electroporation into competent *E. coli* bacteria and plated. Fluorescence was excited with 488 nm light on an LED light table (for one-photon screening) or 1,030 nm using a custom laser scanning system (for 2P screening). The 2P screening system used a 56-W, 160 fs, 1 MHz repetition rate fiber laser (Tangerine HP2, Amplitude Systemes), expanded into a 20 mm long line focus and scanned with a line scanner to illuminate a roughly 2 × 4 cm field of view (FOV) (Supplementary Note 2). In each round, 400 to 1,000 highly fluorescent colonies were picked, and isolated into 96-Well Polypropylene DeepWell blocks (Thermo Fisher Scientific) containing 800 µl of Studier’s autoinduction medium (ZYM-5052; 46). The DeepWell cultures were grown at 30 °C, 250 rpm for 30 h before cells were collected by centrifugation (8,000*g* for 10 min) and rapidly lysed using liquid nitrogen and 40 °C water bath freeze–thaw cycles. Then 100 µl of resulting crude protein extract was transferred to a glass-bottomed plate, in which fluorescence spectra were collected before and after addition of 10 µl of glutamate buffer in PBS (137 mM NaCl, 2.7 mM KCl, 10 mM Na_2_HPO_4_, 1.8 mM KH_2_PO_4_; pH 7.3), using a plate reader (Tecan Infinity M1000). The final glutamate concentration used varied over rounds (200 µM–2 mM) as the affinity of variants under test changed, but were consistent within any given round. In some rounds, multiple final glutamate concentrations were used for lysate measurements to estimate both affinity and dynamic range.

### Protein purification and in vitro characterization

pRSET bacterial expression vector (Thermo Fisher Scientific) encoding the soluble form of the iGluSnFR protein of interest fused to an N-terminal 6-His tag was chemically transformed into *E. coli* strain T7 Express Competent *E. coli* (New England Biolabs). A 4 ml culture containing LB + 100 µg ml^−1^ ampicillin, inoculated with a single bacterial colony, was grown overnight (37 °C, 250 rpm) before being diluted into 500 ml of Studier’s autoinduction medium (ZYM-5052). The culture was grown at 30 °C, 250 rpm for 30 h before cells were collected by centrifugation (8,000*g* for 10 min). The resulting lysate was purified using Ni-NTA chromatography using HisPur Ni-NTA beads (Thermo Fisher Scientific), and dialyzed using Slide-A-Lyzer dialysis cassettes (Thermo Fisher Scientific) with PBS for 24 h. Glutamate-saturated and nominal glutamate-free measurements were performed in 100 mM glutamate in PBS and PBS only, respectively. Glutamate titrations were performed using a series of serially diluted buffers. The fluorescence measurements were fit with a Hill equation: $F_i = F_0 + (F_{\mathrm{max}} - F_0)\frac{{[{\mathrm{Glu}}]^n}}{{K_{\mathrm{d}}^n + [{\mathrm{Glu}}]^n}}$, where *F*_0_ and *F*_max_ are fluorescence without glutamate and with saturated glutamate, respectively, and *n* is the Hill coefficient. Observed apparent Hill coefficients were in all cases 1 ± 0.2. pH titrations were performed by making a series of 100 mM glutamate in PBS buffers and PBS only buffers with varying pH ranging from 5 to 9 (pH adjusted using concentrated HCl and NaOH). Fluorescence intensities as a function of pH were measured in both glutamate-saturated and glutamate-free states, and fitted with a sigmoidal binding function to determine the apparent p*K*a and apparent Δ*F*/*F* as a function of pH. The sigmoidal binding function used was fit using Graphpad Prism v.8 software: $F = F_{\mathrm{min}} + \frac{{F_{\mathrm{max}} - F_{\mathrm{min}}}}{{1 + 10^{({\mathrm{p}}K{\mathrm{a}} - {\mathrm{pH}})}}}$, where *F* is the observed brightness, and the parameters *F*_max_ and *F*_min_ are limit brightnesses at high and low pH, and p*K*a is the apparent p*K*_a_ of the indicator. All terms were free parameters except pH and *F*. Δ*F*/*F*_0_ was computed using the raw fluorescence (*F*) values of glutamate-bound and glutamate-free state with respect to pHs between 5 and 9: $\frac{{F_{\mathrm{bound}}}}{{F_{\mathrm{free}}}} - 1$. Specificity measurements were performed by making buffered solutions in PBS of 20 canonical l-amino acids at 100 mM (pH 7.3), neurotransmitters at 100 mM (pH 7.3), and other drugs at varying concentrations: DNQX (10 µM), D-AP5 (5 mM), DL-TBOA (5 mM), NBQX (5 mM), NMDA (10 mM), CNQX (1 mM) and kainate (5 mM). For l-amino acids and neurotransmitters, fluorescence intensity (excitation 510 nm, emission 540 nm) was measured in the glutamate-free (APO) condition. Δ*F*/*F* was computed by using the following equation: (average fluorescence intensity with l-amino acid/neurotransmitter)/(average fluorescence intensity in apo) − 1. For various drugs, fluorescence intensity (excitation 510 nm, emission 540 nm) was measured in both APO and glutamate-bound (SAT) conditions. Δ*F*/*F* for each drug was independently computed in both APO and SAT conditions using the following equation: (average fluorescence intensity with drug)/(average fluorescence intensity in apo or sat) − 1.

### One-photon photophysical measurements

One-photon fluorescence intensity spectra in presence and absence of glutamate were obtained using the Tecan Infinity M1000 plate reader. Excitation scans were performed from 460 to 530 nm (emission set at 550 nm, bandwidth 10 nm, step 2 nm), emission scans were performed from 500 to 600 nm (excitation set at 480 nm, bandwidth 10 nm, step 2 nm). Fluorescence intensities for glutamate titrations were obtained by exciting at 510 nm and collecting at 530 nm (bandwidth 10 nm). The extinction coefficient was determined using the alkali denaturation method, determining the denatured chromophore concentration using the extinction coefficient of denatured GFP as a reference (44,000 M^−1^ cm^−1^ at 447 nm). The extinction coefficient was computed by dividing the absorbance in nondenatured state by the calculated chromophore concentration. Absolute quantum yield measurements were performed using a Quantaurus-QY spectrophotometer (Hamamatsu Photonics), for purified proteins in PBS (APO condition) and 20 mM glutamate (SAT condition). Quantum yield measurements were made using default settings of the device, exciting between 470 and 510 nm. The quantum yield at 490 nm excitation is reported.

### Stopped-flow kinetics

Measurements of ON kinetics were made using an applied Photophysics SX20 Stopped-flow Reaction Analyzer using fluorescence detection, exciting at 505 nm and detecting with a 520 nm long pass filter, mixing equal volumes of dilute purified indicator protein and PBS + glutamate buffer. Measurements were performed at room temperature (22 °C). Recorded intensity traces were fit in MATLAB; code is available in the code and data package. At least three replicates per concentration were averaged, and a monoexponential saturation curve was fit to the average trace. The duration of the recording fit was from 1 ms (the mixing time of the instrument) to 4 × *t*_90_, where *t*_90_ is the time at which intensity increased to 90% of its maximum (Supplementary Fig. 7). The rate–concentration relationship was then fit to the three-state model using MATLAB’s fit function. Errors of fit were computed from the fit objects using the MATLAB built-in confint function.

### 2P photophysical measurements

The 2P measurements were carried out on protein solutions (2–4 µM) in PBS with 0 mM (apo) and 100 mM (sat) added glutamate, using an inverted microscope (IX81, Olympus) equipped with a ×60, 1.2 NA water immersion objective (Olympus). Excitation was performed with an 80 Mhz Ti-Sapphire laser (Chameleon Ultra II, Coherent) to collect 2P excitation spectra from 710 to 1,080 nm. Fluorescence collected by the objective was passed through a shortpass filter (720SP, Semrock) and a bandpass filter (550BP200, Semrock), and detected by a fiber-coupled Avalanche Photodiode (SPCM_AQRH-14, Perkin Elmer). The brightness spectra were normalized for 1 μM concentration and further used to obtain action cross-section spectra with fluorescein as a reference. FCS was used to obtain the 2P molecular brightness of the iGluSnFR3 soluble proteins at 950 and 1,030 nm, as described in ref. ^46^. Briefly, the peak molecular brightness was defined by the rate of fluorescence obtained per total number of emitting molecules. Here, 50–200 nM soluble iGluSnFR protein solutions were prepared in 100 mM glutamate PBS buffer and excited with 960 and 1,030 nm wavelength at powers ranging from 2–30 mW for 200 s. The obtained fluorescence was collected by an Avalanche Photodiode and fed to an autocorrelator (Flex03LQ, Correlator.com). The obtained autocorrelation data were fit to a diffusion model to determine the number of molecules <*n*> present in the focal volume. The 2P molecular brightness (*ε*) at each laser power was calculated as the average rate of fluorescence *<F*> per emitting molecule *<n*>, defined as *ε* = *<F*>*/*<*n*> in kilocounts per second per molecule.

### Preparation of neuronal culture

Neonatal (P0) Sprague-Dawley (Charles River) rat pups were used regardless of sex. Pups were euthanized, then cortical and hippocampal tissue was dissected and dissociated in papain enzyme (Worthington Biochemicals, roughly 35 U per cortical and hippocampal pair) in neural dissection solution (10 mM HEPES pH 7.4 in Hanks’ Balance Salt Solution) for 30 min at 37 °C. After 30 min, enzyme solution was aspirated out and tissue pieces were subjected to trituration in 10% fetal bovine serum containing modified Eagle medium. Following trituration, cell suspension was filtered through a 40 μm strainer and the resulting single-cell suspension was centrifuged. Cell pellet was resuspended in plating media (28 mM glucose, 2.4 mM NaHCO_3_, 100 µg ml^−1^ transferrin, 25 µg ml^−1^ insulin, 2 mM l-glutamine and 10% fetal bovine serum in modified Eagle medium) and cell counts were taken. Electroporation was conducted using the Lonza/Amaxa 4D nucleofector according to the manufacturer’s instructions, using 500 ng of plasmid and 5 × 10^5^ viable cells per transfection. Transfected cells were then seeded into three replicate wells in poly-d-lysine-coated glass-bottom 24-well plates or 35 mm dishes, and cultured at 37 °C with 5% CO_2_. Cultures were fed twice a week by replacing 50% of the medium with fresh NbActiv.

### Glutamate titrations in neuronal culture

Experiments were performed with rat hippocampal and/or cortical primary cultures as described above. Fourteen days posttransfection, culture medium was exchanged with 1 ml of imaging buffer solution (145 mM NaCl, 2.5 mM KCl, 10 mM glucose, 10 mM HEPES, 2 mM CaCl_2_, 1 mM MgCl_2_, pH 7.3). Glutamate-free apo widefield images were taken at the center of each well using a Nikon Eclipse microscope (×20, 0.4 NA, 505 nm excitation, 525/50 emission). Glutamate-saturated widefield images were taken at the same FOV after the addition of 100 µl of 100 mM extracellular glutamate in imaging buffer (100 mM glutamate, 145 mM NaCl, 2.5 mM KCl, 10 mM glucose, 10 mM HEPES, 2 mM CaCl_2_, 1 mM MgCl_2_, pH 7.3). On-membrane glutamate titrations were performed by exchanging the culture medium with 1 ml of imaging buffer solution containing varying concentrations of glutamate. A scalar constant background was first subtracted from all images. We quantified full-field Δ*F*/*F* for each FOV as: (mean brightness of SAT image)/(mean brightness of APO image) − 1.

### On-cell kinetics in neuronal culture

iGluSnFR3 variants were transfected in mixed hippocampal and neocortical primary cultures as described above. Imaging was performed 13–15 days posttransfection. Culture medium was exchanged with imaging buffer containing 2 µM TTX. Glass pipettes (approximate resistance 5–7 MΩ) were pulled using a P-1000 puller (Sutter instruments). Pipettes were filled with imaging buffer containing 3, 30 or 300 µM Glutamate and connected to a pressure ejector (Picospritzer III; Parker). For each puff, pressure was set to 20 PSI and duration to 3 s. An external trigger was used to initiate a puff after 2 s of baseline imaging. After each puff an intertrial interval was imposed of at least 2 min. Videos were recorded for 7 s at 666 frames per s with 128 × 128 px per frame using a Nikon Eclipse microscope (×20, 0.75 NA, 505 nm excitation center wavelength, 525/50 nm emission band). Recordings were corrected for bleaching by fitting a biexponential curve to the intensity during the 2-s baseline period. Δ*F*/*F* was then computed over the 50-frame period following puff onset, and a region of interest (ROI) selected as a circle of 2 μm radius centered over the pixel with largest ΔFF following Gaussian smoothing in space. Traces were extracted for the ROI in each cell, and a monoexponential saturating curve was fit to each. To remove occasional outliers with slow rates likely due to a misalignment of the pipette and cell, traces with rate constant less than 80% of the median rate constant across cells were discarded.

### Imaging optical minis

iGluSnFR3 variants were transfected in mixed hippocampal and neocortical primary cultures from neonatal (P0) rat pups. Then, 14 days posttransfection, culture medium was exchanged with 1 ml of hyperosmotic buffer containing sucrose (100 mM sucrose, 145 mM NaCl, 2.5 mM KCl, 10 mM glucose, 10 mM HEPES, 2 mM CaCl_2_, 1 mM MgCl_2_, pH 7.3). Next, 2 µM of TTX (diluted in deionized water) was added to each culture medium to silence the firing of APs. Videos were recorded for 60 s at 100 frames per s across three FOV per well using a Nikon Eclipse microscope (×60, 1.4 NA, 505 nm excitation center wavelength, 525/50 nm emission band). Additional experiments (Extended Data Fig. 5 and Supplementary Fig. 1) were performed with a spinning disk confocal microscope (Nikon TiE, Yokogawa CSU-X1) with temperature-controlled sample chamber (37 °C) and focus-locking mechanism for longer-duration recordings. Primary cultures were plated on 35 mm MatTek dishes, and imaged in the same imaging buffer. We recorded for 15 min continuously at each FOV. The recordings were split into 3-min segments due to memory constraints, and processed in a similar manner to the widefield recordings, as described below, with an additional step to remove periodic noise associated with spinning disk illumination.

### Analysis of optical minis

Analysis of optical minis was automated using a constrained nonnegative matrix factorization (NMF) framework with spatial components initialized from a correlation image (inspired by refs. ^47,48^). The NMF model describes a video as a set of spatial footprints, each of which varies over time. The spatial footprints and their activities are all nonnegative. To perform factorization in a memory-efficient manner, each recording was first downsampled twice in each spatial dimension and eight times in time by binning. *F*_0_ was then computed per pixel and subtracted from the video to generate a downsampled baseline-subtracted video. A correlation image was computed where each pixel value is the mean correlation between that pixel and its four-connected neighbors across time, after highpass filtering the video at 8 Hz. NMF was performed on the downsampled baseline-subtracted video using a multiplicative updates algorithm, subject to constraints on the contiguity and sparsity of spatial components. The spatial factors were initialized as 2D gaussians (*σ* = 530 nm) with a spatial extent of 2.8 × 2.8 µm, with no support outside this extent. A factor was added at each local maximum in the correlation image that exceeded a threshold value. An additional 20% of spatial factors were added to model background; these were not spatially constrained during optimization. Following factorization, spatial factors were merged if they were immediately adjacent or overlapping in space and had a Spearman rank correlation in time of at least 0.25. The spatial factors were then upsampled to the original spatial resolution and a final 20 rounds of multiplicative updates were performed on the full-resolution (space and time) data to generate a high-resolution factorization. From the resulting factorizations we computed the SNR and the detected event frequency of each site. The SNR was defined as the amplitude of the third-largest peak in the highpass-filtered signal over the 1-min duration of the recordings used for screening, divided by the standard deviation of noise estimated from the trace power spectrum. Detected event frequency was defined as the number of peaks exceeding three standard deviations of the noise. Detected site density is the number of detected sites with SNR > 3 per unit area.

To produce kymographs (Fig. 3d) and calculate spatial extent of minis (Fig. 3e), the NMF-derived Δ*F*/*F*_0_ time series at each site was highpass filtered and events detected by threshold crossing (three times the standard deviation of the highpass-filtered trace, or 0.2, whichever was greater). The center of each event in time and space was then detected in the original high-resolution video smoothed with a 2.5 pixel Gaussian filter. Events were only retained for analysis if (1) the signal returned to baseline with 3 s, (2) a significant signal (more than 2 s.d. above mean) was observed in the region just outside the smoothing radius (400–800 nm) and (3) the detected peak was the largest signal in the immediate spatiotemporal window (−0.2–0.3 s, 2 μm in space). Because the 1.4 NA objective used has a narrow depth of field, some events are out of focus. To reject out-of-focus events, images were analyzed for spatial extent and the highest-resolution quartile were retained for each condition. Results were similar when retaining the top 50%. Raw images were binned by distance from the spatial center of the event and averaged, then *F*_0_ was calculated as the mean fluorescence within a 200 ms period before event onset, to compute Δ*F*/*F*_0_ kymographs. The distance plot (Fig. 3e) is the mean Δ*F*/*F*_0_ at the frame after event onset for each indicator. The spatial extent *d*_1/2_ was calculated as the distance over which the mean ∆*F*/*F*_0_ dropped below half of the measured maximum (the pixel to which responses were aligned was censored to avoid selection bias). The bootstrap statistic was calculated by randomly resampling each population with replacement and calculating *d*_1/2_ for each group, and comparing the result for the two indicators. See spatialAnalysis.m and summarizeSpatialAnalysis.m on Figshare (10.25378/janelia.21985406)^49^.

### Manipulations of synaptic release

Hippocampal CA1–CA3 regions were dissected with dentate gyrus removed from P1 Sprague-Dawley rats of either sex (mixed litter), dissociated (bovine pancreas trypsin; 5 min at room temperature) and plated on polyornithine-coated coverslips (Carolina Biological; item 633095; 22 × 22 × 0.17 mm borosilicate glass) inside a 6 mm diameter cloning cylinder (Ace Glass) as previously described^50^. Calcium phosphate transfection was performed on 5-day-old cultured neurons with plasmids encoding iGluSnFR variants, or for TeNT experiments, the same concentration of iGluSnFR-encoding plasmid plus a plasmid encoding TeNT^51^. Treatment and control cultures (for example + and −TeNT) for each experiment were paired, that is, used the same batches of neurons, plated and transfected at the same time, and cultured in the same incubator. Experiments were performed at 35 °C using a custom-built objective heater. Coverslips were mounted in a rapid-switching, laminar-flow perfusion and stimulation chamber on the stage of a custom-built laser microscope. The chamber had a width of 8 mm, a length of 10 mm and a height of 2 mm with two platinum electrodes was used for perfusion and stimulation. Then 1 ms pulses of 35 mA were passed across the electrodes to trigger APs. The total volume of the chamber was roughly 150 μl and was perfused at a rate of 400 μl min^−1^. During imaging, cells were continuously perfused in a standard saline solution containing the following in mM: 119 NaCl, 2.5 KCl, 2 CaCl_2_, 2 MgCl_2_, 25 HEPES, 30 glucose, solutions were supplemented with 10 μM 6-cyano-7-nitroquinoxaline-2,3-dione (Alomone) and 5-phosphono-d-norvaline, d-norvaline, 5-phosphono-, d(−)-APV, d-2-amino-5-phosphonovaleric acid, d-2-amino-5-phosphopentanoic acid and d(−)-AP-5 (Alomone). For measuring exocytosis, neurons transfected with iGluSnFr variants were illuminated by a 488 nm laser 2 mW (Coherent OBIS laser) with ZET488/10x and ZT488rdc dichroic (Chroma) through a Zeiss EC Plan-Neofluar ×40 1.3 NA Objective. iGluSnFr fluorescence emission was collected through an ET525/50 m filter (Chroma) and captured with an IXON Ultra 897 electron multiplying charge coupled device (EMCCD) (Andor). iGluSnFr fluorescence was collected with an exposure time of 9.83 ms and images were acquired at 100 Hz. pHluorin-transfected cells were imaged under the same conditions as iGluSnFR variants, except with 8 mW of illumination intensity. Stimulation for firing APs for evoked vesicle fusion were evoked by passing 1 ms current pulses, yielding fields of roughly 12 V cm^−^^2^ using platinum and iridium electrodes. Spontaneous release was easily identified by eye in absence of stimulation. Images were analyzed in ImageJ (http://rsb.info.nih.gov/ij) by using custom-written plugins (http://rsb.info.nih.gov/ij/plugins/time-series.html). GluSnFR measurements were made from manually selected ROI (2 μm diameter) drawn at individual responsive boutons on isolated axons, then averaged across sites for each cell. Boutons were identified as spots that had robust punctate responses to a stimulation train of five field-stimulated APs delivered at 50 Hz (to help find both high and low release probability presynaptic terminals). Fewer than 5% of responsive areas of the axon were discarded as a result of having responsive boutons too close with merging fluorescence responses. This analysis of individual release sites differs from previous analyses of larger areas of mixed neurites^20^. Individual cells were treated as the unit of variability; analysis of variance indicated no effect of culture batch on response amplitudes.

### Neuronal culture screen

Details on culture screening and analysis are provided in Supplementary Note 2. iGluSnFR variants were cloned into a mammalian expression vector and nucleofected into hippocampal and/or cortical primary cultures prepared as described above. Imaging was performed in poly-d-lysine-coated 96-well glass-bottom plates. Fourteen days posttransfection, culture medium was exchanged three times with 500 µl of imaging buffer (145 mM NaCl, 2.5 mM KCl, 10 mM glucose, 10 mM HEPES, 2 mM CaCl_2_, 1 mM MgCl_2_, pH 7.3) and imaged in 75 µl of imaging buffer and drug cocktail to inhibit synaptic transmission (10 µM CNQX, 10 µM (R)-CPP, 10 µM gabazine, 1 mM (S)-MCPG (Tocris)). Neurons were field stimulated with 1 and 20 pulses at 83 Hz, and imaged with a ×10 objective using an EMCCD camera (Hamamatsu Orca Fusion C13440-20C, 1,024 × 1,024, center quad, 5.5 ms exposure, 182 Hz, 798 frames). Illumination was delivered by blue light (470/40 nm, Cairn Research Ltd; emission 525/50 using GFP filter cube). The approximate light density used was 0.34 mW mm^−2^ at 470 nm. Stimulation pulses were synchronized with the camera using data acquisition cards (National Instruments), controlled with Wavesurfer v.1.0.6 software (https://wavesurfer.janelia.org/). Imaging was performed at room temperature. Time-integrated SNR (Fig. 2e) was calculated by integrating the photon response over the duration that it exceeded the baseline (mean response over 80 samples before stimulus onset), and dividing by the shot noise associated with the baseline brightness over that period (that is, the square root of the integrated baseline).

### 2P-excited bleaching measurements in vivo

To investigate photobleaching properties of new iGluSnFR variants in vivo, we imaged dendrites of excitatory cortical neurons in the visual cortex of C57BL/6J mice (Jackson Laboratory), without regard to sex. Viral injections (100 nl, 250 µm below brain surface) in 8-week old mice were performed followed by implantation of a 4 mm cranial window, as described previously^18^. Imaging was performed on a customized AO2P based on a Thorlabs Bergamo II microscope 3–6 weeks after injection. Excitation was provided from a Coherent Discovery NX TPC. The objective was a Olympus XLUMPFLN objective, ×20, 1.0 NA. A FOV of 133 × 67 µm was scanned at 156 Hz for the bleaching experiments. Emitted light was filtered through a 525/50 bandpass dichroic, collected via a ThorLabs PMT2100, and digitized via a laser-clocked National Instruments DAQ card controlled by ScanImage 2020. The imaging parameters were selected to match those we use in typical synaptic imaging experiments. Recordings of 12,000 frames were collected at 156 fps with laser power of 33 mW at sample at a wavelength of 1,010 nm, maximum depth of 100 µm below dura for both SF-Venus.iGluSnFR.A184V and iGluSnFR3.v857-PDGFR. Recordings were motion corrected^52^ and pixels were selected that exceeded a threshold mean brightness over time. The traces for the selected pixels were then averaged to generate a single photobleaching trace. A constant background level, the mean recorded brightness in an unlabeled portion of the image, was subtracted for each FOV. A total of 34 FOV (from five mice) for v857 and 27 FOV (from five mice) for SF-Venus.iGluSnFR.A184V were analyzed.

### One-photon in vivo imaging

We performed bulk imaging of iGluSnFR responses in the same mice transduced with AAVs encoding iGluSnFR variants as described for 2P-excited bleaching measurements, above. Four animals per construct were imaged at four time-points after surgery (3, 4, 5 and 6 weeks postinjection). In every animal there were four injection sites, two for a given iGluSnFR3 construct and two for SF-Venus.iGluSnFR.A184V serving as a within-animal control. A custom-build widefield microscope was used, incorporating an Orca Flash 4.0 Camera and a Thorlabs ITL200 tube lens. Each mouse received four nonoverlapping AAV injections. Low-magnification fluorescence recordings of all injection sites were performed with an Olympus UPlanApo ×4 objective, and sites were recorded individually through a ×25 Olympus XLPlan N 1.05 NA objective. Excitation: SOLIS 470 nm LED; hq 470/40 41109 Chroma Excitation filter. Et 560/40 319882 Chroma Emission filter. 512 × 512 16-bit images (four-times pixel binning in both *x* and *y*) were acquired at 100 fps using Hamamatsu HC Image software. Illumination intensity was constant across all sessions. Drifting grating visual stimuli were presented using the same apparatus, grating spatial and temporal frequencies, and anesthesia conditions described in ref. ^18^. Motion stimuli (2 s duration) of eight directions were presented in sequence spaced by 2 s periods of luminance-matched gray screen, eight times each.

Recordings were motion corrected^53^ and background was subtracted by manually selecting a ROI within vasculature in each FOV. Pixels of interest were automatically detected based on their brightness and distance from automatically detected vasculature. Pixels of interest were averaged to generate a single trace, separated into low-frequency (*F*_0_) and high-frequency (Δ*F*) components, and motion artifacts were then subtracted from the Δ*F* trace by regression of the low-pass filtered *x* and *y* offsets obtained during motion correction (and corresponding quadratic terms) against the trace. Responses were averaged across stimulus presentations to generate a direction-independent mean response for each session. A subset of sessions showed very weak or no evoked modulation. We therefore sorted sessions for each site by power in the (0.2 0.3) Hz frequency interval and used only the top 50% of sessions per variant to calculate the grand mean response for each indicator.

Intrinsic signal imaging (ISI) was performed during the first and last imaging sessions for each animal to assess visual responsiveness at each recorded site over time. ISI was performed on the same microscope as fluorescence imaging, using a Mitutoyo 7.5 × 0.21 NA objective, by collecting reflected light from a M625L4 LED (Thorlabs) illuminating the FOV from an angle outside the acceptance cone of the objective. This commonly used geometry for ISI reduces collection of light reflected from glass surfaces. Then 16-bit full-field images were collected at 33.3 fps using Hamamatsu HC Image software. Light intensity was set to the maximum that avoided camera pixel saturation anywhere within the FOV. The same eight stimuli used for fluorescence imaging were repeated over a period of approximately 30 min to generate ISI traces, which were then processed in a similar manner to fluorescence recordings.

### Mouse preparation for AO2P imaging

WT mice (C57BL/6J, males, younger than 8 weeks old, Jackson Laboratory) were used for thalamocortical bouton imaging. Adult (more than 8 weeks old) transgenic mice (Scnn1a-Tg3-Cre, no. 009613, Jackson Laboratory) were used for layer four dendritic spine imaging. Mice were anesthetized with isoflurane using a precision vaporizer, 3% (vol/vol) in oxygen for induction and 1–2% (vol/vol) for maintenance and given the analgesic buprenorphine subcutaneously (0.1 µg per gram body weight). Body temperature was maintained at 37 °C with a heating pad during anesthesia. The animal was placed in a stereotaxic frame. A 4-mm craniotomy was created over the right vS1 cortex (centroid at 1.5 mm posterior to the bregma and 3.4 mm lateral from the midline). The dura was left intact. A cranial window by a single 4-mm round coverslip (no. 1) was embedded in the craniotomy and sealed with cyanoacrylate glue (Loctite, catalog no. 401). Meta-bond (Parkell) was further applied around the edge to reinforce stability. A titanium head-bar was attached to the skull with Meta-bond and the remaining exposed bone was covered with dental acrylic (Lang Dental).

Virus injection was conducted before the craniotomy was made. To label the thalamocortical projections in vS1 cortex, Cre-dependent SF-Venus-iGluSnFR.A184V/SF-iGluSnFR.A184V/iGluSnFR3.v82.GPI/iGluSnFR3.v857.GPI/iGluSnFR3.v857.PDGFR (AAV2/1.hSyn.FLEX.SF-Venus-iGluSnFR.A184V, original titer 3.6 × 10^13^ GC ml^−1^; AAV2/1.hSyn.FLEX.SF-iGluSnFR.A184V, original titer 1.12 × 10^13^ GC ml^−1^; AAV2/1.hSyn.FLEX.iGluSnFR3.v82.GPI, original titer 8.46 × 10^13^ GC ml^−1^; AAV2/1.hSyn.FLEX.iGluSnFR3.v857.GPI, original titer 3.17 × 10^13^ GC ml^−1^; AAV2/1.hSyn.FLEX.iGluSnFR3.v857.PDGFR, original titer 2.56 × 10^13^ GC ml^−1^) and AAV2/1.hSyn.Cre virus (original titer 3.1 × 10^13^ GC ml^−1^) were diluted and mixed to the final titer of from 8.5 × 10^12^ to 1.1 × 10^13^ GC ml^−1^ for iGluSnFR virus and 3.4 × 10^10^ or 6.3 × 10^9^ GC ml^−1^ for Cre virus and then injected (50 nl, 10 nl min^−1^) to the barreloids of VPM nucleus in WT mice, 1.7 mm posterior to the bregma, 1.8 mm lateral from the midline and at a depth of 3.25 mm. To label the layer four dendrites and spines in vS1 cortex, virus of iGluSnFR3.v857.GPI (AAV2/1.hSyn.FLEX.iGluSnFR3.v857.GPI, original titer: 3.17 × 10^13^ GC ml^−1^) was diluted to 1.06 × 10^13^ GC ml^−1^ and injected at a 45° angle into the vS1 of Scnn1a-cre mice to the target coordinates of 1.5 mm posterior to bregma, 3.4 mm lateral from midline and 400 µm deep. For all injections glass pipettes (Drummond) were pulled and beveled to a tip at 30-µm outer diameter and a syringe pump (Kd Science, Legato 185) was used to control the infusion.

### In vivo AO2P imaging

In vivo imaging was carried out after 4 weeks of expression and 3 days of habituation for head fixation. All imaging experiments used head-fixed awake mice under AO2P. Mice were anesthetized with isoflurane shortly and given a retro-orbital intravenous injection of 20 µl Cy5.5-dextran PBS solution to label the lumen blood vessels 30 min before imaging. AO correction was applied at the imaging depth below 350 µm using the method described previously^33^. Briefly, direct wavefront sensing was performed through the labeled microvessels within a square region of 50 to 100 µm on edge, with the exact central location as the functional imaging ROI. The excitation wavelength for wavefront sensing was 1,250 nm. The measured wavefront error was then applied to the deformable mirror during the functional imaging of the same ROI. Wavefront measurement and correction were repeated when switching to a different ROI. For functional imaging, the laser was tuned to 950 nm for SF-iGluSnFR.A184V, 1,030 nm for SF-Venus-iGluSnFR.A184V and iGluSnFR3.v82.GPI and 1,000 nm for iGluSnFR3.v857.GPI/PDGFR. Postobjective power was under 100 mW for all measurements.

### Vibrissa tracking and stimulation

The vibrissae of the mice were trimmed 3 days before functional experiments, leaving only one vibrissa, that was C1, C2 or D1, whose corresponding cortical column had an optimal expression of iGluSnFR. The mice were head fixed to the imaging rig with a running disk. The experiments were carried out in the absence of visible light and the running disk was illuminated by two infrared LEDs (Thorlabs, M940L3) to generate a bright-field contrast for vibrissae tracking. A high-speed camera (Basler, acA1300-200um) was used to track the vibrissae at the frame rate of 500 Hz. Air-puff deflection was used for vibrissa stimulation. Pulse-controlled compressed air, 20-ms pulse width, 5 p.s.i. at the source, was delivered through a fine tube, which was placed parallel to the side of the mouse snout and 20 mm away from the targeted vibrissa. The frequency of the air puffs was from 2 to 30 Hz. A motorized moving pole was used for dynamic pole touch experiments. The pole moves back and forth within a 5-mm range along the azimuthal direction at an average speed of 1.25 mm s^−1^. The positions of the vibrissa and pole were extracted using DeepLabCut^54^ and custom code written in MATLAB.

### Single-cell electroporation and 2P imaging in mouse V1 in vivo

For in vivo experiments, 50–55-day-old male C57Bl/6 mice were implanted with a headplate for subsequent 2P glutamate imaging. Anesthesia was induced with 2% isoflurane in pure oxygen and maintained at 1.5% during surgery. The body temperature was continuously monitored and maintained by a heating plate at 37.5 °C. Both eyes were covered with ophthalmic ointment. After injecting a local anesthetic (2% xylocaine) and an analgesic (Metamizole, 200 mg kg^−1^), a headplate was attached to the skull above the left hemisphere of the brain with dental cement.

The procedure for single-cell electroporation of plasmids was adopted from a previously described method with some modifications^55^. The plasmid of either SF-iGluSnFR.A184S or iGluSnFR3.v857 was dissolved in an artificial intracellular solution (135 mM K-gluconate, 4 mM KCl, 10 mM HEPES, 4 mM Mg-ATP, 0.3 mM Na_2_-GTP, 10 mM Na-Phosphocreatine) at a final concentration of roughly 100 ng μl^−1^. A fluorescent dye (100 μM OGB-1) was also included to visualize the patch-pipette and to verify the success of electroporation. For electroporation, a 3 mm round craniotomy was made above the left primary visual cortex (V1) and a coverslip of a similar size was fixed onto the craniotomy with Vetbond (3M). The glass coverslip had a small perforation that allowed the access of a patch-pipette to the cortical tissue underneath. After electroporation, the perforated coverslip was replaced by an intact one and the edge of the craniotomy was completely sealed with Vetbond.

Imaging of labeled neurons was performed 7 days after the electroporation with a custom-built 2P microscope. The microscope was equipped with a 12-kHz resonant scanner (Cambridge Technology) and controlled by LabVIEW. Excitation laser was a mode-locked Ti:sapphire laser (Mai Tai DeepSee, Spectra-Physics) and the average power below the objective (×40, NA 0.8, Nikon) was between 20 and 30 mW. Dendrites of neurons expressing SF-iGluSnFR.A184S were imaged at 100 Hz frame rate with 940 nm excitation light and dendrites labeled with iGluSnFR3 were imaged at 200 Hz with 950 nm excitation light. For visual stimulation, a 10.5-inch Samsung tablet was placed at a distance of 10 cm in front of the right eye of the mouse. The screen covered a visual space of 96° in azimuth and 70° in elevation and had a mean brightness of 4.8 cd m^−^^2^. Full-field square wave drifting gratings (0.03 cycle per degree spatial frequency, 2 Hz temporal frequency, 2 s duration) were presented at eight directions (45° step, in a fixed order) using a custom Android app.

For simultaneous 2P axonal imaging and cell-attached recordings, a *z*-stack of images of the labeled neuron was acquired and the axon was manually traced to identify unambiguously boutons from the same neuron. Before recording, a perfusion chamber was fixed on top of the headplate, and the coverslip on the cortex was changed to one with an opening. Warm artificial cerebro-spinal fluid (125 mM NaCl, 26 mM NaHCO_3_, 4.5 mM KCl, 2 mM CaCl_2_, 1.25 mM NaH_2_PO_4_, 1 mM MgCl_2_ and 20 mM glucose) was perfused throughout the experiment to keep the brain temperature constant. A patch-pipette filled with artificial cerebro-spinal fluid containing 25 μM Alexa Fluor 594 was approached under visual control to the soma of the labeled neuron and, once the tip of the patch-pipette touched the membrane, gentle negative pressure was applied to obtain a seal resistance of 40 MΩ. Once a stable cell-attached recording was established, spontaneous activity was recorded, while 2P glutamate imaging of axonal boutons was performed at a frame rate of 500 Hz. Electrophysiological signals were acquired either in the current-clamp or in the voltage-clamp mode by a patch-clamp amplifier (EPC10, HEKA).

### Mouse V1 data analysis

All data were processed and analyzed using MATLAB. Drifts of images in the *xy* plane were corrected using a cross-correlation based registration algorithm. ROI were automatically segmented based on the correlation image for each recording. The mean fluorescence signal *F* from each ROI was extracted and the fluorescence change was calculated as ∆*F*/*F*_0_ = (*F* − *F*_0_)/*F*_0_. For dendritic recordings with visual stimulation, *F*_0_ was defined as the mean of fluorescence signals during a period of 2 s before visual stimulation. For spontaneous activity recorded from axonal boutons, *F*0 was defined as the mean of fluorescence signals during all ‘No AP periods’. ‘No AP periods’ were the intervals between APs, starting 200 ms after the last AP. All traces displayed in Fig. 5 were filtered using a Savitzky–Golay filter.

For the analysis of kinetics and specificity (Fig. 5e), the traces from axonal boutons were denoised with a nonnegative deconvolution algorithm^56^ using the estimated range of tau for each sensor. ‘Single APs’ were APs that were separated by more than 200 ms from the preceding and following APs. The amplitude of a ‘single-AP event’ was the peak fluorescence value during a period of 20 ms following the electrically recorded spike. ‘No AP events’ were local maxima of the deconvolved trace during ‘No AP periods’.

Pixelwise orientation maps (Fig. 5i,j) were generated as previously reported^18^. Briefly, responses (mean fluorescence change) to eight visual stimulation directions were obtained for each pixel. The responses were mapped onto orientation vector space and summed. The preferred orientation was the angle of the resultant vector. Orientation selectivity was calculated as the length of the resultant vector divided by the sum of responses to each orientation. The response magnitude was defined as the two-norm of responses to all directions. The hue of the orientation map encodes preferred orientation, the saturation corresponds to orientation selectivity, and the lightness is set by response magnitude and is normalized to the pixel with the largest magnitude from the map of v857.

### Expansion microscopy

Female Emx1-cre mice 8 weeks old were injected in cortex at multiple sites in a 3.5 mm craniotomy over V1 with 40 nl of 5 × 10^11^ GC ml^−1^ AAV2/1 encoding hSyn.Flex-iGluSnFR.v857 in the three backbones (PDGFR, GPI and SGZ; one construct per mouse; two mice per construct). A cranial window was affixed to seal the craniotomy and allow expression to be confirmed before histology. Mice were transcardially perfused 16 days after virus injection, and gels prepared from labeled cortical regions using a protease-free variant of Ten-fold Robust Expansion (TREx) microscopy^32^. Gels were labeled with primary and secondary antibodies as follows: Primary: mouse anti-Bassoon no. ab82958 (abcam) dilution 1:200, rabbit anti-Homer no. ab97593 (abcam) dilution 1:200, chicken anti-GFP no. ab13970 (abcam) dilution 1:500. Secondary: goat anti-mouse AF594 A-11012 (Invitrogen), goat anti-rabbit Atto 647N 40839-1ML-F (Sigma Aldrich) and goat anti-chicken AF 488 A-11039. For imaging, gels were expanded in purified water (Millipore Milli-Q) for 1 h, placed in a glass-bottomed dish (coated in poly-l-lysine to prevent sample drift), and imaged using a Zeiss LSM800 confocal microscope using a ×40, 1.1 NA water immersion objective. All images were acquired with the same image settings, except photomultiplier voltages were adjusted to prevent digitizer saturation and adjust dynamic range across samples. Synapses were annotated manually by Fourier transform, and quality of annotations was verified blind to genotype by KP. The annotator was asked to: ‘Draw a line connecting the presynapse to the postsynapse on an axis locally perpendicular to the iGluSnFR-labeled membrane, in the 2D image plane corresponding to the center of intensity of the pre- and postsynaptic labels. Only synapses with their axis in the plane of the image should be labeled.’ Annotations, image data and software to visualize them (requires MATLAB) are available on Figshare (10.25378/janelia.21985406).

### Statistics and reproducibility

Values and errors reported throughout the text are mean ± s.e.m. unless otherwise noted. Statistical analyses were performed in Graphpad Prism v.8 (in vitro photophysics) and MATLAB (others), as described in the text. Data and code used to produce the figures, and instructions for generating them, are provided on Figshare (10.25378/janelia.21985406). Representative example experiments were performed the following number of times independently with similar results to those shown: Fig. 3a, 18 cultures; Fig. 3g, three cultures (nine cells); Fig. 4c, six mice; Fig. 5a,b, five neurons and Supplemental Fig. 7, three cultures.

### Reagent availability

A variety of mammalian and bacterial expression plasmids for iGluSnFR3 variants v82 and v857 are available through Addgene (https://www.addgene.org/browse/article/28220233/). Requests for AAVs and other reagents can be made by contacting K.P.

### Reporting summary

Further information on research design is available in the Nature Portfolio Reporting Summary linked to this article.

## Online content

Any methods, additional references, Nature Portfolio reporting summaries, source data, extended data, supplementary information, acknowledgements, peer review information; details of author contributions and competing interests; and statements of data and code availability are available at 10.1038/s41592-023-01863-6.

### Supplementary information

Supplementary Information Supplementary Notes 1–3, Figs. 1–7 and Tables 1 and 2.

Reporting Summary

Supplementary Video 1 Widefield recording of optical minis using iGluSnFR3.v857.GPI in mixed hippocampal and neocortical primary cultures 14 days posttransfection. Culture medium was exchanged with hyperosmotic buffer containing 100 mM sucrose, and APs were silenced using 2 µM TTX. Video was recorded at 100 frames per second using a Nikon Eclipse microscope (×60 1.4 NA).

Supplementary Video 2 In vivo 2P recording of spontaneous activity (no visual stimulus) in excitatory cortical neurons in mouse visual cortex using iGluSnFR3.v857.PDGFR using a custom 2P microscope (×20, 1.0N A, Exc - 1010 nm, Em 525/50, 156 Hz, 122 × 61 µm FOV).

Supplementary Video 3 Averaged images of thalamocortical boutons with sparse labeling of iGluSnFR3.v857.PDGFR in L4 during whisker stimulation. Images were acquired at the frame rate of 247.9 Hz for 60 s and interpolated to 500 Hz. The 60-s recording was then averaged 60-fold to produce the 1-s average recording shown. Images are acquired at the depth of 350 µm below the pia with a FOV of 50 × 25 µm.

Supplementary Video 4 Averaged images of dendritic spines labeled with iGluSnFR3.v857.GPI in L4 during whisker stimulation. Images were acquired at the frame rate of 130 Hz for 60 s and interpolated to 500 Hz. The 60-s recording was then averaged 60-fold to produce the 1-second average recording shown. Images are acquired at the depth of 365 µm below pia with a FOV of 50 × 30 µm.

## Figures and Tables

**Extended Data Table 1:**
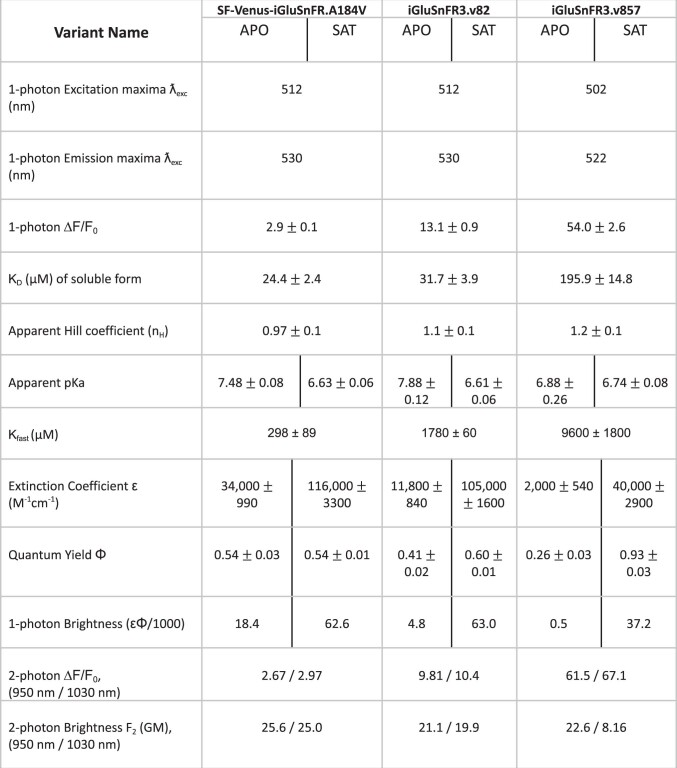
Photophysical characterization of WT (SF-Venus-iGluSnFR.A184V), iGluSnFR3.v82, and iGluSnFR3.v857

All measurements are made with purified protein. N = 3, mean ± s.e.m.

## Data Availability

The data used to produce the figures are provided on Figshare: 10.25378/janelia.21985406. GenBank accession numbers for iGluSnFR3 variants are OQ656874–OQ656880.

## Code availability

The code used to produce the figures are provided on Figshare: 10.25378/janelia.21985406.
