# Supplementary material for: Glutamate indicators with improved activation kinetics and localization for imaging synaptic transmission
Source: Nat Methods. 2023 May 4;20(6):925–34. doi: 10.1038/s41592-023-01863-6 (PMC10250197; doi:10.1038/s41592-023-01863-6)
Supplement: Supplementary file 1 — Supplementary Notes 1–3, Figs. 1–7 and Tables 1 and 2. [file 41592_2023_1863_MOESM1_ESM.pdf]

nature methods

Article

https://doi.org/10.1038/s41592-023-01863-6

Glutamate indicators with improved activation kinetics and localization for imaging synaptic transmission

In the format provided by the authors and unedited

Supplement to:

Glutamate indicators with improved activation kinetics and localization for imaging synaptic transmission

Abhi Aggarwal[1,2], Rui Liu[3], Yang Chen[4], Amelia J Ralowicz[5], Samuel J Bergerson[5], Filip Tomaska[1, 6], Boaz Mohar[1], Timothy L Hanson[1], Jeremy P Hasseman[1], Daniel Reep[1], Getahun Tsegaye[1], Pantong Yao[7], Xiang Ji[3], Marinus Kloos[4], Deepika Walpita[1], Ronak Patel[1], Manuel A Mohr[1,8], Paul W Tilberg[1], The GENIE Project Team[1], Loren L Looger[1,9], Jonathan S Marvin[1], Michael B Hoppa[5], Arthur Konnerth[4], David Kleinfeld[3,10], Eric R Schreiter[1], Kaspar Podgorski[1,2,*]

Affiliations

1. Janelia Research Campus, Howard Hughes Medical Institute, Ashburn, Virginia, USA 20147
2. Allen Institute for Neural Dynamics, Seattle, Washington, USA 98109
3. Department of Physics, University of California, San Diego, La Jolla, California, USA 92093
4. Institute of Neuroscience and Cluster for Systems Neurology (SyNergy), Technical University of Munich, 80802 Munich, Germany
5. Department of Biological Sciences, Dartmouth College, Hanover, New Hampshire, USA 03755
6. Department of Physiology, Second Faculty of Medicine, Charles University, Prague, Czech Republic
7. Neurosciences Graduate Program, University of California San Diego, La Jolla, California, USA 92093
8. Department of Biosystems Science and Engineering, Eidgenössische Technische Hochschule (ETH) Zurich, 4058 Basel, Switzerland
9. Howard Hughes Medical Institute, Department of Neurosciences, University of California, San Diego, La Jolla, California, USA 92093
10. Section of Neurobiology, University of California, San Diego, La Jolla, California, USA 93093

* Correspondence should be addressed to K.P. (kaspar.podgorski@alleninstitute.org)

Supplementary Notes

Supplementary Note 1. Kinetic effects on the spatial specificity of neurotransmitter indicators

We set out to develop iGluSnFR variants that better distinguish synaptic versus extra-synaptic glutamate signals. To explore how kinetic properties of the indicator impact these signals, here we consider a simple reaction-diffusion system in which glutamate transients are released from point sources, and glutamate subsequently interacts with the expressed indicator. Consistent with our data and previous work (e.g., (56)), we model the indicator as having three states, described below. We model the extracellular interface as a 2D surface.

We define the *spatial specificity* of a signal as the ratio of a signal near the release site (at distance r=50 nm) to that farther away (r=1 μm). We first consider the spatial specificity of the glutamate concentration itself. For a non-cooperative indicator, the spatial specificity of the ligand (i.e. glutamate) is an upper bound on the spatial specificity of the indicator (i.e. iGluSnFR). This is because a non-cooperative indicator has a linear or sublinear response to its ligand, meaning that a proportional increase in ligand results in at most a proportional increase in indicator signal. Since the ligand concentration close to the release site is always larger than that further away, the ratio of the indicator signal near the release site to that farther away is at most equal to the ratio of the concentrations (Figure SN1-1a).

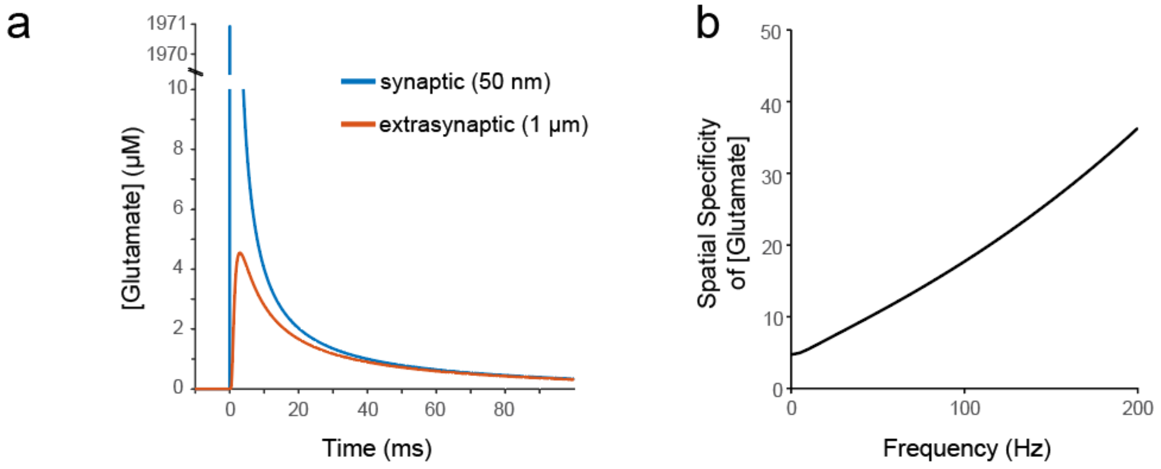

Figure SN1-1. Simulation of glutamate kinetics. a) Simulated glutamate concentration at the synapse (50 nm from release site) and outside the synapse (1 μm from release site) in the absence of buffering. b) The ratio of the amplitude spectra of the signals in (a). This is the spatial specificity of glutamate as a function of temporal frequency. The spatial specificity of glutamate is an upper bound on the spatial specificity of a non-cooperative glutamate indicator.

The spatial specificity of the glutamate concentration can be characterized in the frequency domain (Figure SN1-1b). Faster components of the time-varying glutamate concentration have higher spatial specificity than slower components. To better reproduce features of the glutamate signal that distinguish synaptic from extrasynaptic transients, an indicator should have fast rise and decay times, the imaging rate should be fast enough to capture those components, and data analysis should appropriately filter for higher-frequency components of the signal. At low frequencies, all indicators will show relatively low spatial specificity, because the glutamate concentration itself has low spatial specificity at those frequencies. This reasoning motivated the use of ΔF_{fast} as a screening criterion in neuronal culture (Supplementary Note 2).

iGluSnFR and its variants exhibit saturating ON rates, in which the formation of fluorescent species cannot exceed a maximum rate v_{max} due to a rate-limiting conformational change. ON rate saturation is characteristic of a kinetic model with at least 3 states, in which ligand binding (k_{+1}) rapidly forms a bound but dim species (S1), followed by a slower transition (k_{+2}) to a bright state (S2). This three-state model exhibits two different kinds of saturation phenomena. ON rate saturation occurs when large concentrations of ligand are rapidly applied, and unbound indicator (S0) becomes depleted in favor of bound-but-dim indicator (S1). This is distinct from saturation of the fluorescent state (S2), which occurs on slower timescales and involves depletion of the S1 state.

Certain parameters of the above model can be inferred experimentally from stopped-flow measurements in which known concentrations of glutamate are rapidly mixed with small amounts of unbound indicator (Figure SN1-2), and the rate at which resulting fluorescence approaches steady state (ON rate; k_{obs}) is measured. In the model, k_{+1} and k_{-1} are assumed to be large compared to k_{+2} and k_{-2}, so the equilibrium between S0 and S1 (governed by the dissociation constant $K_{fast}=k_{-1}/k_{+1}$) is achieved much more rapidly than that between S1 and S2. Applying the steady-state approximation for the first equilibrium, the ON rate expressed as a function of [glutamate] is a saturating function:

$$k_{obs}= (k_{+2}[Glu]/K_{fast})/(1+[Glu]/K_{fast}) + k_{-2}$$

K_{fast} is the glutamate concentration that half-saturates the ON rate. The ON rate at saturating [Glu], v_{max}, is ($k_{+2}+k_{-2}$), and the y-intercept of the ON rate is k_{-2}.

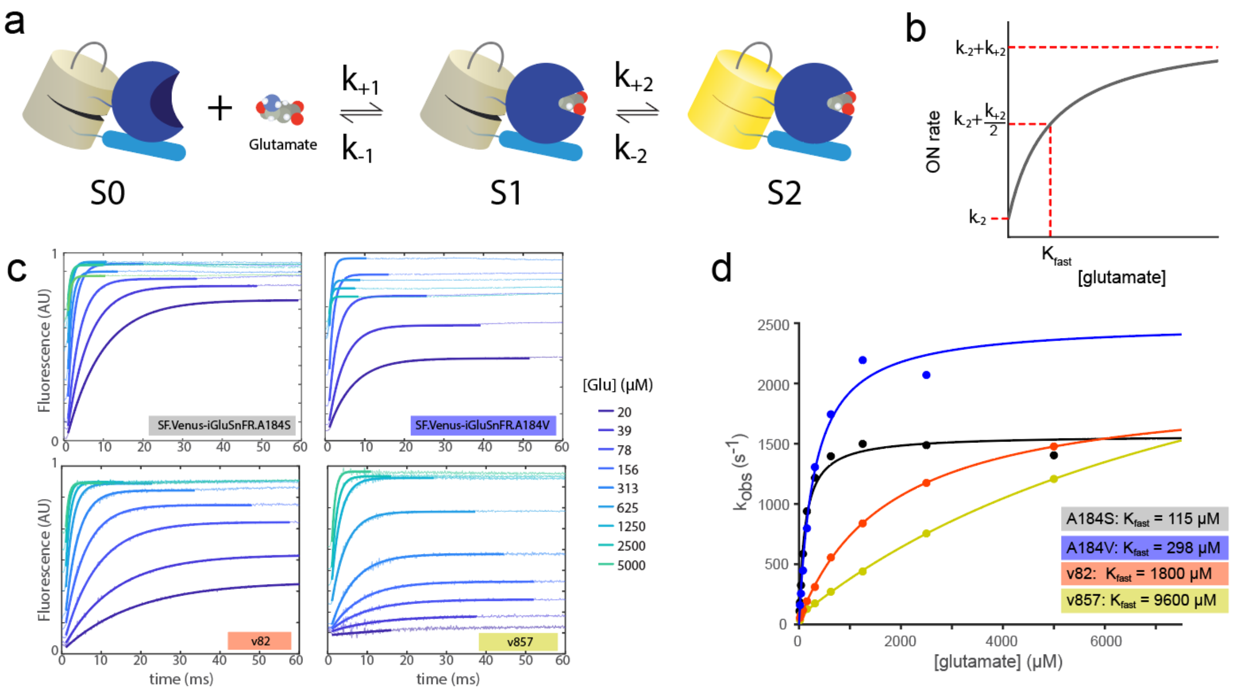

Figure SN1-2. Model and measurements of iGluSnFR kinetics. a) Three-state model of iGluSnFR kinetics. In this model, initial glutamate binding is followed by a second, rate-limiting transition to the fluorescent state. b) Schematic of the ON rate in the three-state model as a function of ligand concentration. K_{fast} is the rate-half-saturating ligand concentration. c) Stopped-flow measurements of initial rates for purified soluble iGluSnFR mixed with equal volume glutamate buffer to the specified final [glutamate]. Thin lines are raw measurements, thick lines are corresponding single-exponential fits. The dead time of the instrument is approximately 1 millisecond. The decreased amplitude at the highest glutamate concentrations observed for A184V and A184S is typical of these variants. d) Measured initial rates (dots) for SF-iGluSnFR (A184V, A184S) and iGluSnFR3 variants at different glutamate concentrations and corresponding fits to the three-state model. Measurements were made in purified soluble protein at room temperature. The inferred value of K_{fast} for v857 is outside the tested range making the inferred value of this parameter uncertain.

	iGluSnFR (2013)	SF-Venus-iGluSnFR.A184V	SF-Venus-iGluSnFR.A184S	iGluSnFR3.v82	iGluSnFR3.v857
K_{fast}	846 ± 294 μM	298±89 μM	115±27 μM	1.78 ± 0.06 mM	9.6 ± 1.8 mM
k_{-2}	n.m.	43.9 ± 4.4 s^{-1}	25.1±18.4 s^{-1}	36.1 ± 1.2 s^{-1}	34.1 ± 2.4 s^{-1}
k_{+2}	1286 ± 200 s^{-1}	2502 ± 162 s^{-1}	1569±86 s^{-1}	1955 ± 23 s^{-1}	3331 ± 421 s^{-1}

Table SN1-1. 3-state model kinetic parameters of select iGluSnFR variants. All values are minimum MSE fits (95% confidence interval) to stopped-flow data using purified protein. For v857, the fit K_{fast} is beyond the range of

concentrations tested; K_{fast} and k_{+2} for this indicator are therefore poorly constrained by the data. k_{-2} is fit to low-concentration kinetic data (Figure SN1-4).

We varied K_{fast}, k_{+2} and k_{-2} in our simulation and assessed their effects on spatial specificity (Figure SN1-3). K_{fast} was the most important parameter for improving spatial specificity in these simulations. ON rate saturation is severe when the instantaneous glutamate concentration exceeds K_{fast}. Because instantaneous glutamate concentrations are much greater within the synapse than outside it, low values of K_{fast} reduce the spatial specificity of the indicator (Figure SN1-3). Large values of K_{fast} are therefore desirable for high spatial specificity.

Increasing k_{+2} increases k_{obs}, and produces a higher bright state fraction for a brief impulse of glutamate, but does not improve spatial specificity at frequencies relevant for imaging experiments (Figure SN1-3). The rate constant k_{-2} controls the duration of response to a brief impulse of glutamate but does not affect spatial specificity of a rate-limited indicator.

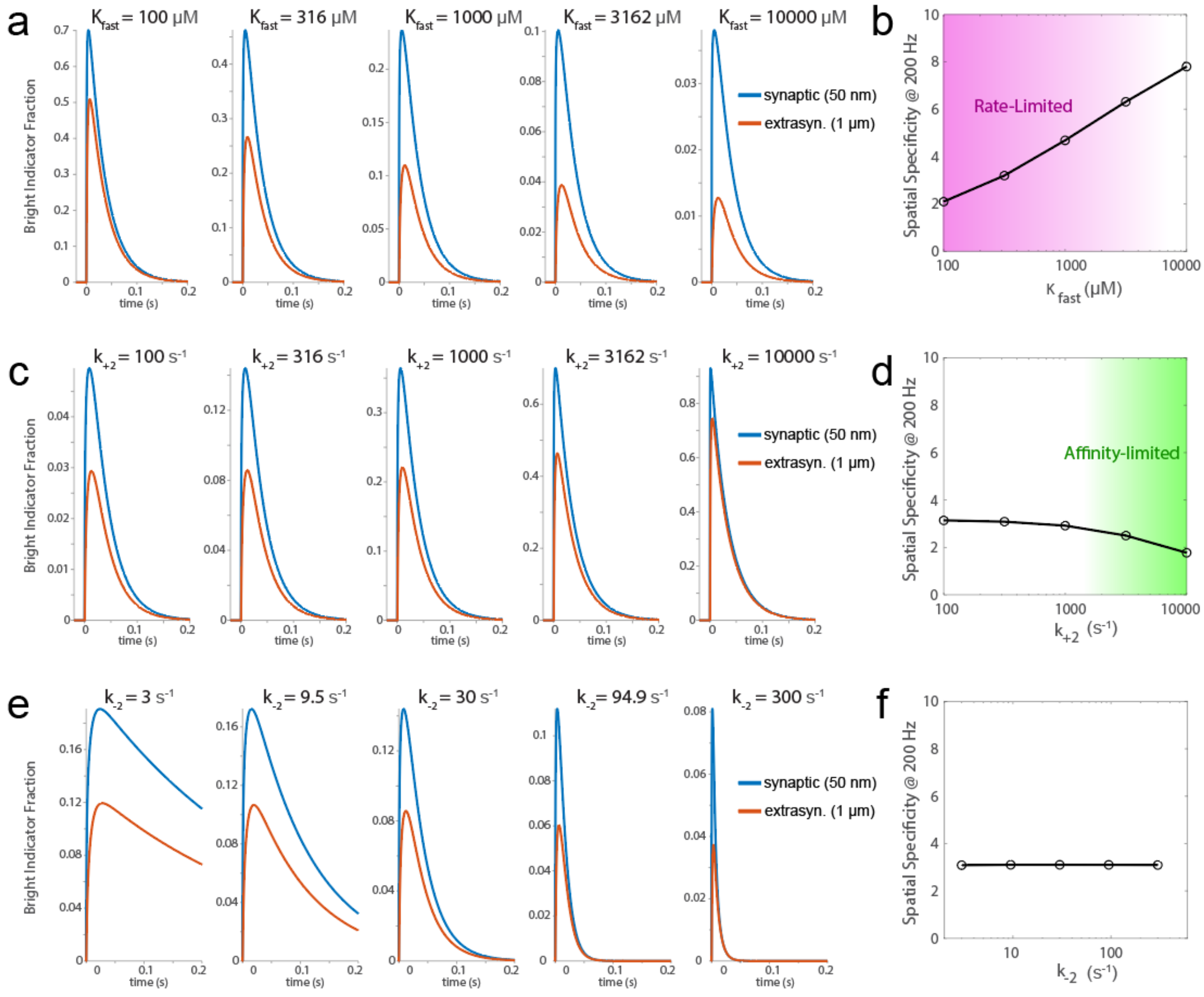

FIgure SN1-3. Effects of kinetic parameters on spatial specificity. a) Simulated fluorescence responses within and outside the synapse while varying K_{fast}. b) Spatial specificity at 200 Hz for different values of K_{fast}. The spatial specificity in the shaded region is limited by saturation of the S0-S1 transition ("kinetic saturation"). c) Simulated fluorescence responses within and outside the synapse while varying k_{+2}. d) Spatial specificity at 200 Hz for different values of k_{+2}.The spatial specificity in the shaded region is limited by saturation of the S2 state ("affinity saturation").e) Simulated fluorescence responses within and outside the synapse while varying k_{-2}. f) Spatial specificity at 200 Hz for different values of k_{-2}.The spatial specificity in the shaded region is limited by the slow response of the indicator.

The apparent affinity of the indicator, K_D, depends on k_{+2}, k_{-2}, and K_{fast}:

$$K_D=K_{fast}k_{-2}/(k_{-2}+k_{+2})$$

At low values of K_D, saturation of the bright state S2 can occur (Figure SN1-3c). This is a second form of saturation (distinct from kinetic saturation) that can in principle reduce spatial specificity ('affinity-limited' indicators in SN1-3d). However, experiments in neuron culture suggest that existing iGluSnFR variants are not affinity-limited: SF-iGluSnFR variants do not approach their maximum brightness in any measurable volume (even at single pixels) during field stimulation experiments, as evidenced by subsequent buffer exchange with saturating glutamate (20). Therefore, the spatial specificity of these indicators is not limited by their affinity.

The above measurements and simulations suggest that the spatial specificity of existing iGluSnFR variants is limited by ON rate saturation, which is determined by K_{fast} and varies substantially among existing variants (22,56). We therefore set out to develop new iGluSnFR variants that are capable of reporting glutamate at high temporal frequencies, are not rate-limited (high K_{fast}), have large responses to brief glutamate impulses, and remain not affinity-limited.

Simulation Methods. Simulations were performed using finite differencing methods in 2 dimensions, to model the geometry of the intercellular space. The MATLAB code for the simulations is available in the software package as `\Figure 1 and Supplementary Note 1\kinetics modeling\glutamateDiffusion.m`. The simulations reflect general principles of neurotransmitter sensing. Parameters used were selected to be relevant for glutamate sensing but are not meant to precisely model a particular indicator. Ligand reuptake and endogenous buffering are not modeled. The buffering effects of the indicator are modeled but not discussed here, for a discussion see (42).

References

56. Coates C, Kerruth S, Helassa N, Török K. Kinetic Mechanisms of Fast Glutamate Sensing by Fluorescent Protein Probes. Biophys J. 2020 Jan 7;118(1):117–27.

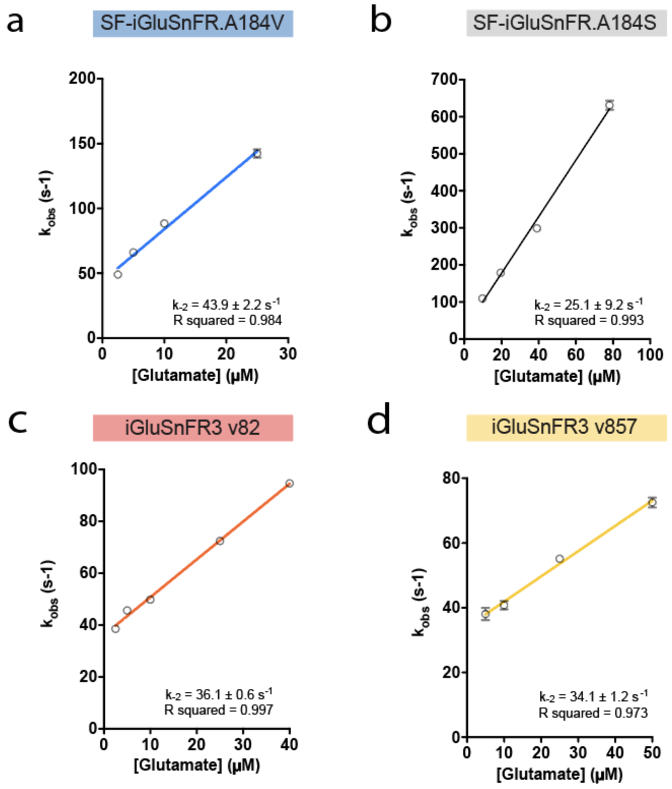

FIgure SN1-4. Stopped-flow kinetic measurements of iGluSnFR variants at low [glutamate]. Kinetics of glutamate binding by stopped-flow fluorescence spectroscopy for a) SF-iGluSnFR.A184V, b) SF-iGluSnFR.A184S, c) iGluSnFR3 v82, and d) iGluSnFR3 v857. Equal volumes of 2 μM protein and glutamate (various concentrations) were mixed at 1:1 ratio using a stopped-flow spectrometer (Applied Photophysics). Each data point is k_{obs} determined using pseudo-first order analysis (n=3 stopped flow replicates of a single protein sample). The observed rates are fit using a linear regression and k_{-2} is determined using the y-intercept. Error bars represent SEM.

Supplementary Note 2. Directed evolution and multi-assay screen of iGluSnFR3

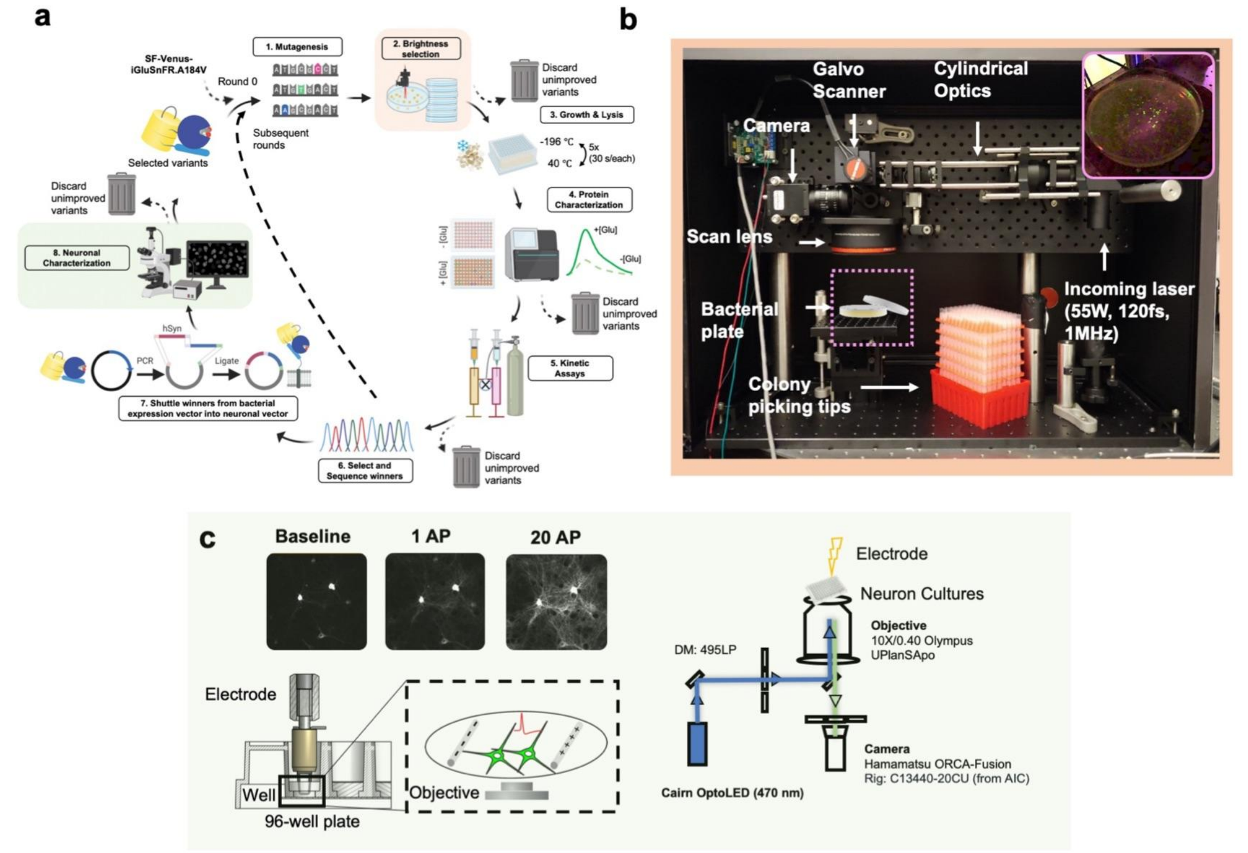

Figure SN2-1. a) Schematic of multi-assay directed evolution screen. The smaller loop (steps 1-6) was performed for 20 generations, followed by the larger loop (steps 1-8) for an additional 2 generations. b) Custom large-area two-photon excitation system used to select colonies for 2P-excited brightness. Inset shows an example plate with fluorescent colonies excited by the system. The infrared excitation beam appears as magenta in this photo, but is invisible to the human eye. c) Schematic of in-neuron culture screening using the GENIE Project apparatus. Top, example frames showing a variant's baseline and responses to 1 AP and 20 AP in the culture assay. Bottom, primary cultures were plated in a 96-well imaging plate, imaged using an inverted microscope and stimulated by a motorized dipping electrode (reprinted from (24)). Right, schematic of the culture assay imaging path. Schematic in (a) was created using BioRender.com (2021).

We began by subjecting WT (Round 0; **Figure SN2-1a**) to error prone polymerase chain reactions (EP-PCR; mean rate 4.5 to 9 DNA substitutions/clone) to generate mutant libraries of the soluble iGluSnFR coding sequence that were then assembled into a pRSET plasmid vector (Step 1). These libraries were transformed into electrocompetent *E. coli*, and plated onto agar plates containing ampicillin. In each round, we screened roughly 50 agar plates (~10^5 colonies) using either 2-photon (1030 nm) or 1-photon excitation (488 nm), and selected the brightest colonies by eye (Step 2) through

laser-safety glasses. Macroscopic 2-photon excitation (~2x4 cm) was achieved using a low repetition rate, high-power femtosecond fiber laser (1 MHz repetition rate, 55 W average power, 120 fs pulsewidth; Tangerine HP2, Amplitude Systemes) that was shaped into a line focus and scanned along one dimension at 500 Hz using a galvanometer mirror scanner (**Figure SN2-1b**). The selected bacterial colonies were inoculated into autoinduction medium, incubated overnight, and washed with repeated cycles of centrifugation, decanting, and resuspension in glutamate-free PBS to remove glutamate. Bacteria were lysed using freeze-thaw cycles of liquid nitrogen (-196 °C) and warm water bath (40 °C) (see methods) (Step 3). Bacterial lysis by other means (i.e., chemically using B-PER, mechanical disruption, or sonication) resulted in lower protein yield, higher assay variability, and/or reduced $\Delta F/F_0$.

Clarified bacterial lysates were used for multiple assays (Step 4). In all rounds, a plate reader was used to measure fluorescence excitation spectra before and after the addition of glutamate. In most rounds, glutamate was added to a final concentration of 500 uM, but after the E23D mutation became fixed in our variant pool, we used 200 uM [glutamate] to select for higher-affinity variants. In lysate assays, variants were selected on the basis of the combination of $\Delta F/F_0$, excitation spectrum (we selected for variants with red-shifted peak excitation relative to EGFP), and glutamate-bound brightness in the plate reader assay (which may reflect intrinsic brightness of the indicator variants, but also properties such as maturation rate and bacterial copy number). DNA was then isolated and sequenced for the selected variants, and the rest were discarded (Step 6).

Following the first round of selection, the retained population of variants were recombined through staggered extension PCR (StEP) (44), then subjected to the same screening and selection process (Steps 2 to 6). Mutagenesis in subsequent rounds proceeded using diversification (via EP-PCR, site-saturation, site-directed mutagenesis, or combinatorial mutagenesis) and recombination, in alternating rounds.

From the third round onward, we performed additional assays in lysate and purified protein to further remove undesired variants from the pool. In lysate, we performed pH titrations in the presence and absence of glutamate, discarding variants with high pKa in the glutamate-bound state. In purified protein, we measured 2-photon excited molecular brightness at 1000 nm excitation, discarding variants with low brightness, and we measured *in vitro* kinetics in a stopped-flow assay to select for variants with high v_{max} and non-saturating k_{obs} (indicative of high K_{fast}) (Step 5). Additionally, we performed glutamate titrations, and evaluated extinction coefficient, quantum yield, and molecular brightness in glutamate-free and glutamate-bound states. In each case, the sequential selection we performed ensured that all retained variants jointly met all selection criteria.

After 20 rounds of in vitro selection, a library of 120 variants were subcloned into a neuronal surface display vector (Step 7). These were then characterized in cultured rat neurons (Step 8) using a modified form of the established GENIE Project pipeline for high-speed imaging of field-stimulated neuron cultures in the presence of post-synaptic receptor antagonists to control the number and timing of evoked action potentials (24). This first neuronal screen identified v82 and several related variants as

high-sensitivity indicators with similar on-neuron kinetics to WT. The soluble forms of these variants were further diversified and screened in bacteria and lysate, for brightness, response amplitude, and spectral properties. 812 variants were selected from this pool, generated as membrane-tagged mammalian expression plasmids, and screened in a second round of neuronal culture screening. After the second round of screening, the assay was repeated for selected variants with additional stimulus conditions (1,5,10,20,160 APs; Figure 2 of main text).

For neuronal culture screening, the GENIE Project apparatus was modified to use a high-speed camera (180fps, Hamamatsu ORCA Fusion) and to automatically focus on labeled neurites at the surface of the coverslip for each well. Each variant was transfected 4 times and plated into 16 wells. Each well was recorded while stimulating 1 and 20 AP field stimuli. We characterized a variety of properties for each well, including baseline brightness, photostability, and for both 1 AP and 20 AP conditions: the number of responsive pixels, the fractional change in brightness ($\Delta F/F_0$), SNR, and response onset and offset kinetics (Figure SN2-2). Summary data and code used for analysis is included in the data supplement.

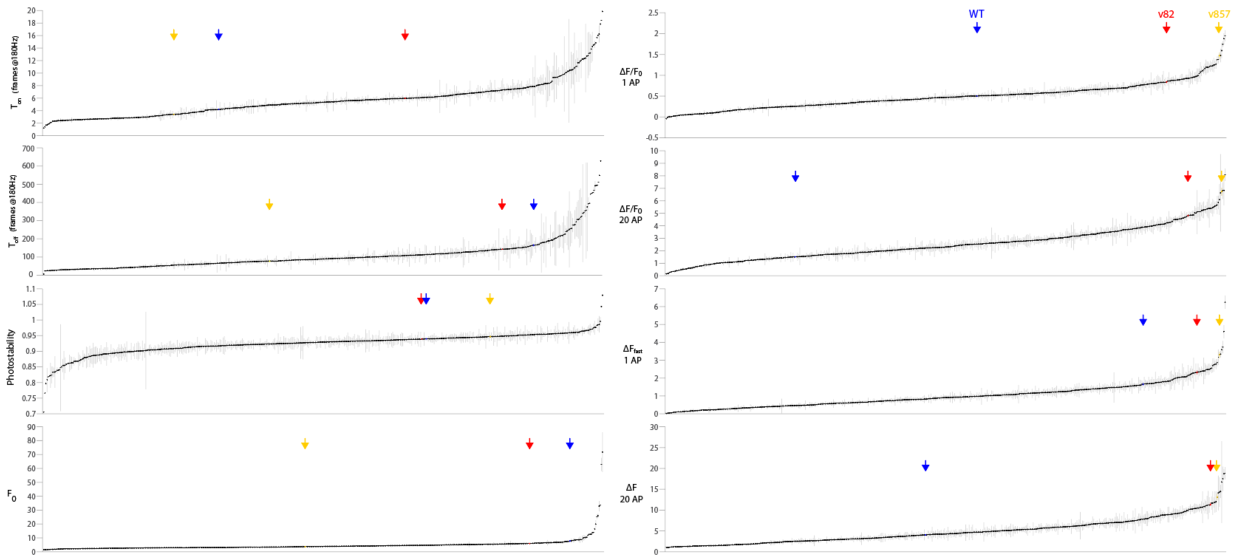

Figure SN2-2. Properties of variants measured in the second round of neuron culture screen. Dots denote individual variants, sorted according to the plotted value. 499 variants are plotted; an additional 310 variants did not meet the threshold for minimum number of significantly responding pixels per well (300 labeled pixels exceeding a Z-score of 3.5). Error bars denote standard error of the mean across wells (N=14-16 wells per variant). Blue, Red, and Yellow arrowheads (and corresponding colored data points) denote values for WT, v82, and v857 variants, respectively. ΔF_{fast} (arbitrary units) is proportional to the excess number of photons collected in a 6-frame window following the stimulus in the top 10% of responding pixels. T_{on} is the calculated 10-90% rise time of the 1AP response, fit with sub-frame resolution using linear interpolation. v857 was selected in this screen on the basis of its desirable combination of properties. Photostability was calculated as the fraction of original intensity retained following 2 seconds of illumination; it is a measure of rapid photobleaching and/or photoswitching.

An important criterion we used to select variants in the cultured neuron assay was ΔF_{fast}, the excess number of photons collected per pixel within 33 ms (6 frames) of 1AP stimulation, compared to a baseline before stimulation, averaged over the top 10% most responsive pixels in the same time window that met a minimum brightness threshold. These 10% most responsive pixels were selected using a separate stimulus presentation to avoid selection bias. The motivation for this criterion was to enrich measurements for synaptic glutamate signals. We reasoned that an indicator with increased spatial specificity at synapses should show large signals at a fraction of labeled pixels (those close to release sites), and would potentially show smaller signals at the remaining pixels. Moreover, this spatial difference should be most apparent over a short period of time following release (see Supplementary Note 1). Example responses for several variants are plotted in Figure SN2-3, for both the selected 10% of pixels (solid lines), and for all responding pixels (dashed lines). A large difference between the solid and dashed traces indicates that a subset of highly responsive pixels exhibit larger responses than the mean labeled pixel, which is expected for a more spatially specific indicator.

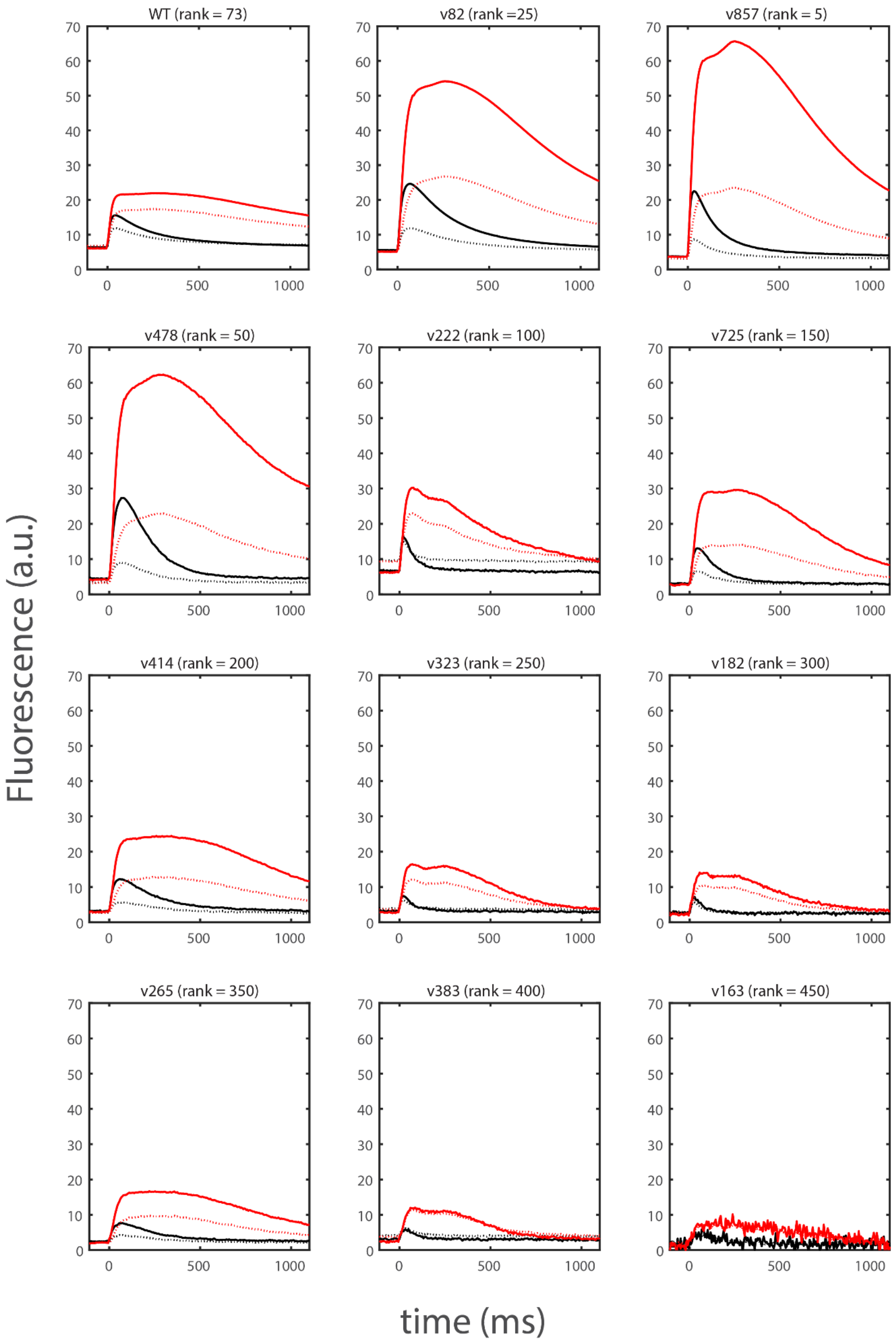

Figure SN2-3. Example traces for variants measured in the second round of neuron culture screen. The black and red traces denote responses to 1 and 20 APs, respectively. Solid and dashed traces denote mean responses of the top 10% of pixels selected from each well (as described in text) and all labeled pixels, respectively. The rank of each variant in ΔF_{fast} within the screening round is displayed. The displayed traces show ~1 second of the 5-second total recording duration.

Supplementary Note 3. Mutations present in iGluSnFR3 variants

Variant v82 has 13 mutations relative to WT (with positions numbered from the opening of the iGluSnFR coding sequence[1]): E23D, N33S, Q35H, D43E, F82Y, V182S, N196D, S252N, N254I, R273H, A332T, I334N, T335S. Variant v857 has 15 mutations relative to WT (mutations relative to v82 are underlined): E23D, N33S, Q35H, D43E, F82Y, V182S, N196D, S252N, <u>N254T</u>, R273H, A332T, I334N, T335S, <u>L395F</u>, <u>G414S</u> (Figure SN3-1). The sites E23, V182, and G414 have been identified in previous studies (19,20,22).

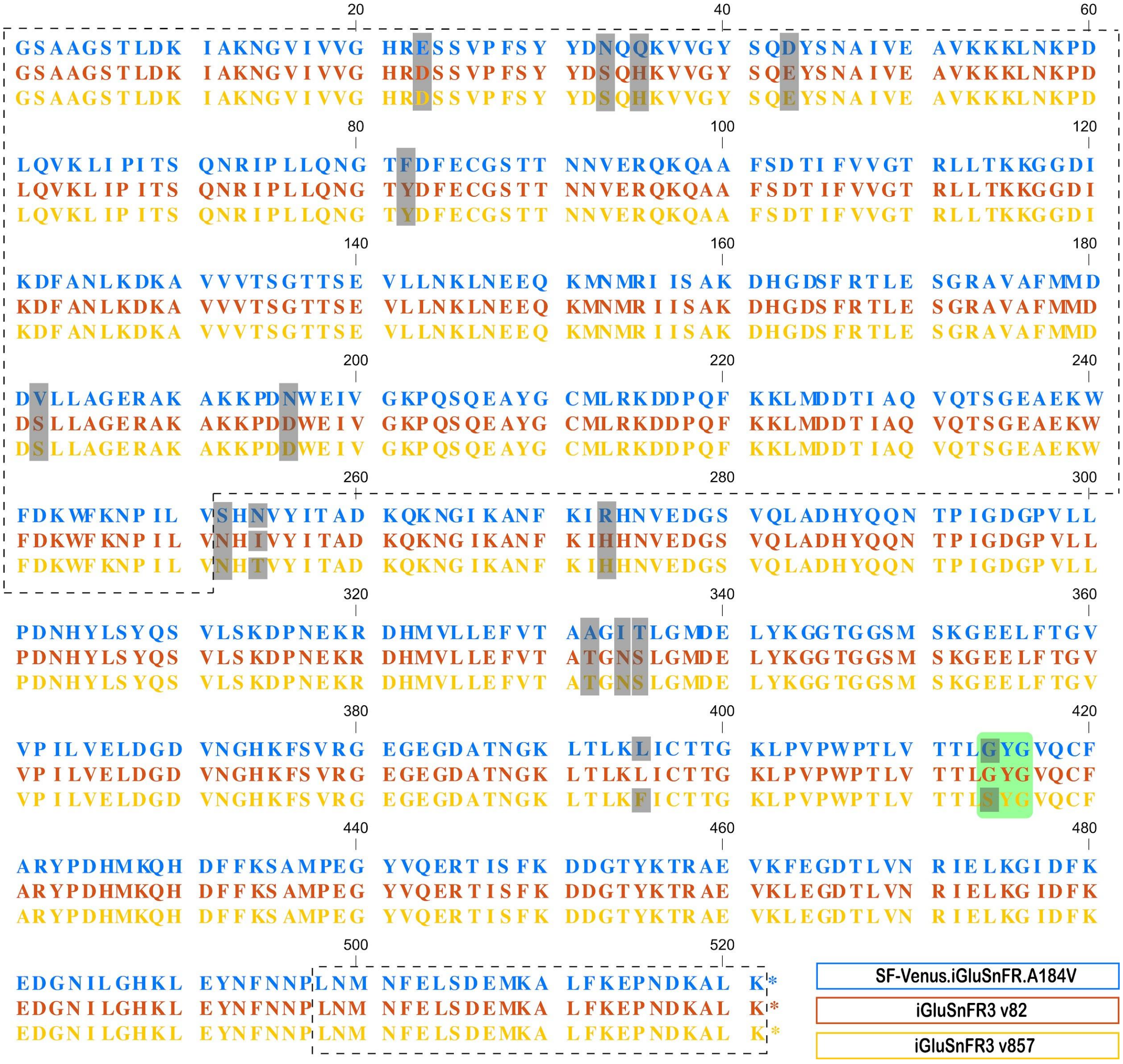

Figure SN3-1. Sequence alignment of WT (blue), v82 (red), and v857 (yellow). Grey highlights denote mutations with respect to WT. Green box highlights the chromophore. Dotted outlines denote regions derived from gltI.

[1] This numbering scheme uses position indices 2 lower than those used by Marvin et al 2013

Notably, even though mutagenesis was performed across the entire coding sequence, most of the mutations selected by screening lie near a putative interface between iGluSnFR's circularly-permuted fluorescent protein domain and the large (N-terminal) fragment of gltI (Figure SN3-2). No functionally-relevant point mutations were identified in the small (C-terminal) α-helix fragment of gltI. Truncation of this fragment in soluble iGluSnFR v857 at residue 498 yielded a functional sensor, indicating that this domain is not necessary for glutamate binding or fluorescence change.

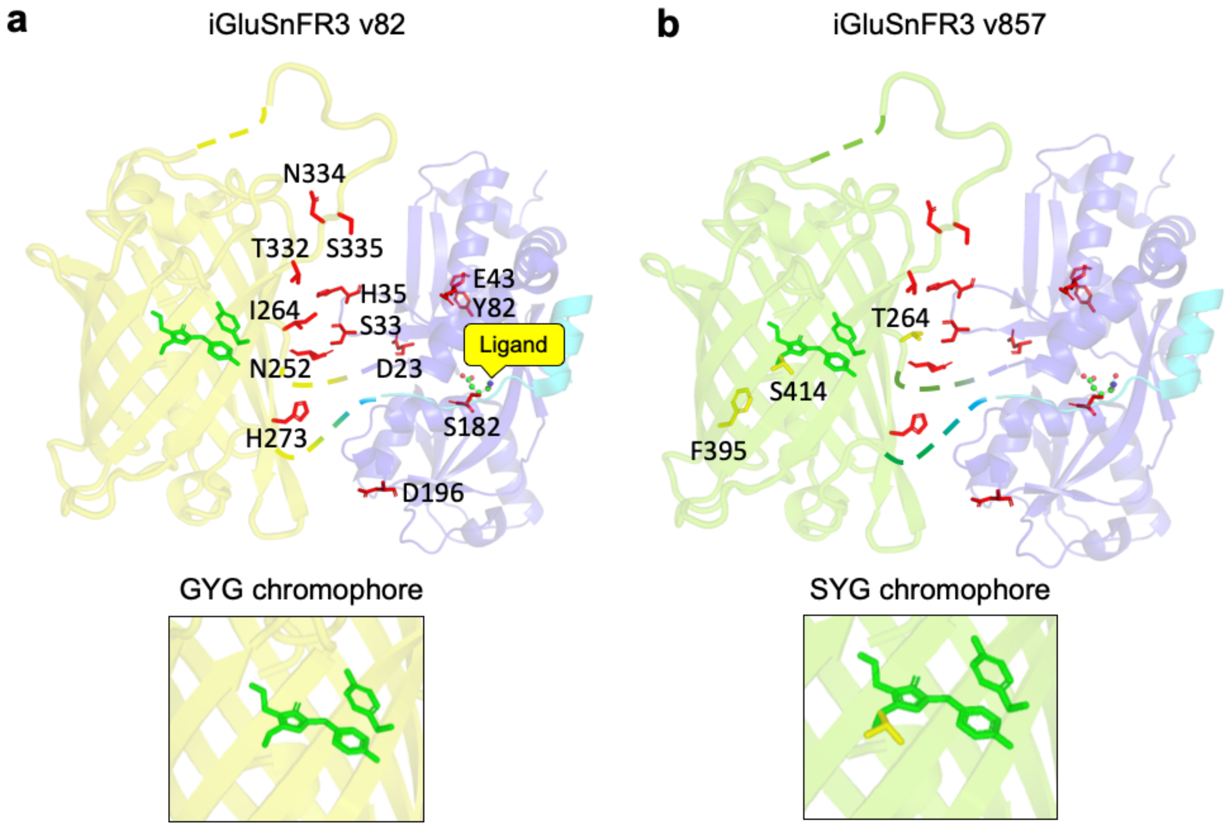

Figure SN3-2. Crystal structures of YFP (PDB: 1F0B) and gltI (PDB: 2VHA) overlaid with mutations present in a) v82 and b) v857. Mutations are shown in red sticks. Zoom in boxes show the distinct chromophores of v82 and v857. In this cartoon, the structures of YFP and GltI have been placed side-by-side in the orientation that best aligns the termini that are joined in iGluSnFR. The precise fold and orientation of these domains in iGluSnFR is unknown, but we assume the fold is similar given the retained functions of the protein, and the orientation is constrained by the short linkers joining them.

The above mutations were identified over multiple rounds of screening involving multiple assays. Early rounds of screening relied exclusively on random mutagenesis and StEP recombination and resulted in a population of variants with high brightness, high $\Delta F/F_0$ and micromolar K_D containing the mutations E23V,

N33S, Q35H, D43E, P75Q, F82Y, N196D, S252N, R273H, A332T, I334N, T335S, F463L. The variants selected at this point exhibited low maximum maximum ON rates (k_{+2}) (Figure SN3-3).

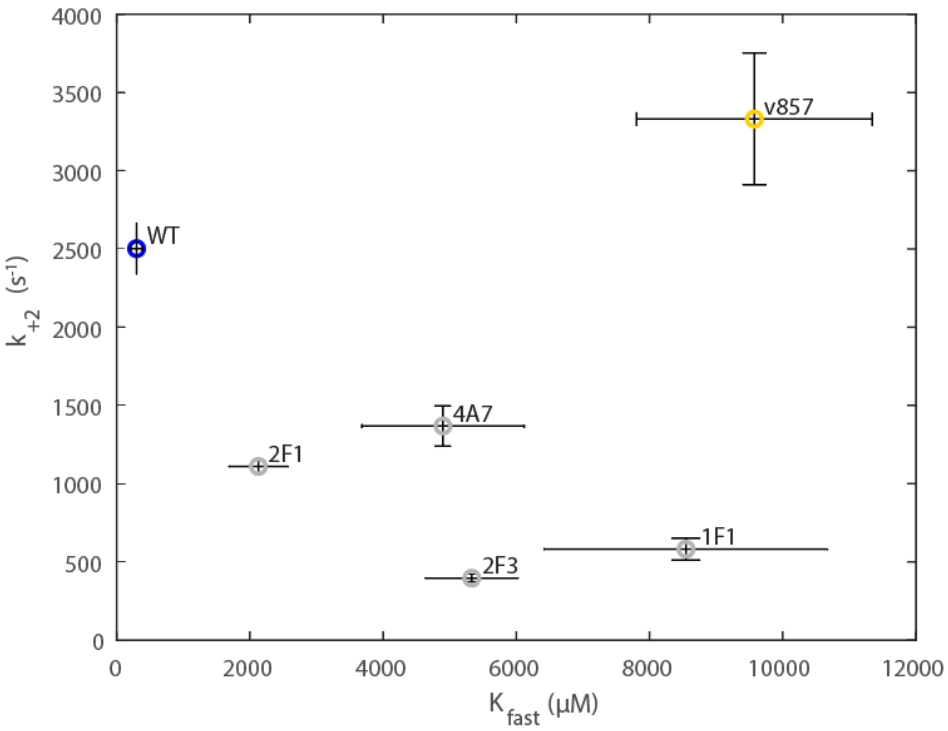

Figure SN3-3. Kinetic parameters of several high ΔF/F_0 variants identified through random mutagenesis, prior to rational mutagenesis of E23 and A182 and neuron culture screening. WT and v857 variants are shown for comparison. Data points denote least squares fit. Errorbars denote 95% confidence interval of the fit. Data from 3 stopped-flow time series at each of 8 concentrations. v1F1: [WT + E23V N33S Q35H D43E F82Y N196D N254I R273H A332T G333T I334N T335S F463L]. v2F1: [WT + E23V N33S Q35H D43E F82Y N196W N254I R273H A332T I334N T335S F463L]. v2F3: [WT + E23V N33S Q35H D43E F82Y N196D M224I N254I R273H A332T G333Q I334N T335S F463L]. v4A7: [WT + E23V N33S Q35H D43E F82Y N196D S252N N254I R273H A332T I334N T335S F463L].

To diversify kinetic properties and identify variants with large values of both k_{+2} and K_{fast}, we then rationally mutated sites previously shown to affect iGluSnFR kinetics: E23 (1), and S70 (1, 2), on top of the selected population. We produced 40 variants mutated at these two sites, which we characterized in purified protein using a stopped-flow kinetics assay. In these variants, E23D improved the maximum ON rate, while E23V, E23A, and E23K improved sensitivity but harmed ON rate. Mutants of this pool at position S70 did not result in any fluorescent bacterial colonies. The E23D mutation reduced the affinity of the soluble sensor to an undesirable degree. We therefore further diversified this pool by adding the affinity-increasing mutation A182S (A184S in (20)) to E23D-containing variants. In soluble protein, we found that addition of A182S to E23D variants increased their affinity approximately 10-fold, without substantially altering ON rates. The resulting population of variants formed the first pool (n=120 variants)

screened in neuron culture. We found that variants containing both E23D and A182S mutations showed the largest rapid-onset (0-20 ms post-stimulus) responses to 1 AP field stimuli, our performance criterion in the neuron culture screen (Supplementary Note 2). The best of these was the variant v82. Further diversification, consisting of saturation mutagenesis at sites selected for in earlier rounds of screening and error-prone PCR, resulted in identification of the additional mutations N254T, L395F, G414S present in v857. Kinetic characterization of v857 confirmed this variant's high values of k_{+2} and K_{fast} (Figure SN3-3), in addition to other desirable properties identified in the multi-assay screen (Figures 1-3).

To assess functional relevance of the 15 mutations in v857, we individually reverted each single amino acid mutation to its WT identity. We evaluated each reversion in purified soluble protein, on the basis of excitation and emission spectra, *in vitro* $\Delta F/F_0$ to saturating glutamate, and affinity (Table 1). In all cases, reversion decreased *in vitro* $\Delta F/F_0$, or in case of S182V, resulted in no fluorescence in the purified protein. Reversion of the chromophore mutation (S414G) resulted in a bathochromic shift, while the remaining positions had no effect on the excitation or emission maxima. The effects of G414S in v857 are consistent with previous work (57) finding that GFP derivatives with the SYG chromophore have increased quantum yield and blue-shifted excitation spectrum relative to GYG. The above results suggested to us that all of the accumulated mutations in v857, when assessed independently and assayed *in vitro*, are necessary for optimal function of the sensor.

We created a database of mutations identified throughout our multi-assay screen, with measurements of $\Delta F/F_0$, $K_{D,}$ k_{obs}, with the parameters measured in bacterial lysate or in purified protein. These measurements are available at the GitHub repository: https://tlh24.github.io/glusnfr-analysis/.

References

57. Jung, G., Wiehler, J. & Zumbusch, A. The Photophysics of Green Fluorescent Protein: Influence of the Key Amino Acids at Positions 65, 203, and 222. *Biophysical Journal* 88, 1932–1947 (2005).

Table SN3-1. Measurements of all single-residue reversions of iGluSnFR v857 in soluble purified protein. NF = non-fluorescent in bacteria. Values at mean ± SEM. n = 3 for each measurement.

Mutant	Exc_{max} (nm)	Em_{max} (nm)	$\Delta F/F_0$	K_D (µM)
SF-Venus-iGluSnFR.A184V	512	530	2.9 ± 0.1	24.4
iGluSnFR3 v857	502	522	54.0 ± 2.6	195.9
iGluSnFR3 v857 + D23E	502	522	17.5 ± 0.3	70.9
iGluSnFR3 v857 + S33N	502	522	13.6 ± 0.6	307.3
iGluSnFR3 v857 + H35Q	502	522	39.5 ± 1.3	180.1
iGluSnFR3 v857 + E43D	502	522	10.7 ± 0.1	106.4
iGluSnFR3 v857 + Y82F	502	522	2.7 ± 0.2	60.0
iGluSnFR3 v857 + S182V	NF	NF	NF	NF
iGluSnFR3 v857 + D196N	502	522	5.5 ± 0.5	137.3
iGluSnFR3 v857 + N252S	502	522	10.6 ± 0.2	271.6
iGluSnFR3 v857 + T254N	502	522	11.4 ± 0.8	164.8
iGluSnFR3 v857 + H273R	502	522	28.6 ± 1.1	238.5
iGluSnFR3 v857 + T332A	502	522	4.2 ± 0.2	117.9
iGluSnFR3 v857 + N334I	502	522	18.1 ± 0.6	70.6
iGluSnFR3 v857 + S335T	502	522	13.6 ± 1.3	24.9
iGluSnFR3 v857 + F395L	502	522	15.9 ± 0.7	172.5
iGluSnFR3 v857 + S414G	510	524	7.0 ± 0.3	66.0

Supplementary Figures

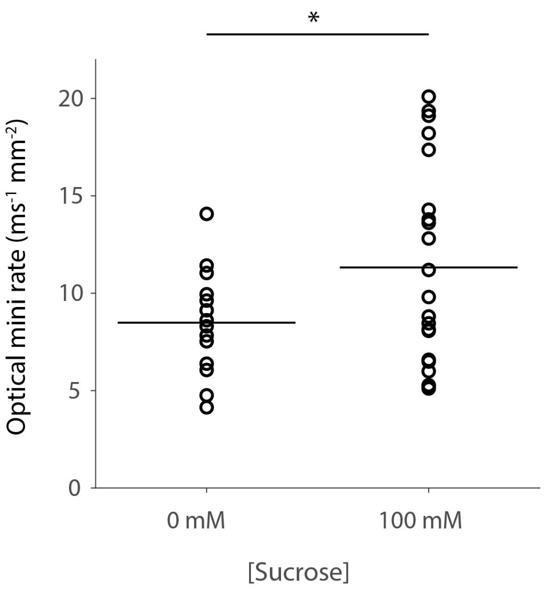

Supplementary Figure 1. Effect of osmolarity on optical mini rate in primary cultures expressing iGluSnFR3.v857. Rate of detected optical minis in imaging buffer before (left) and after (right) addition of an equal volume of 200 mM sucrose in imaging buffer. These recordings were performed at 37°C using a spinning disk confocal microscope (compare vs. room temperature widefield imaging, Fig 3). N=14 FOVs (0 mM), 21 FOVs (100 mM). Horizontal lines denote the mean for each condition. *:$p<0.05$, unpaired two-sided 2-sample t-test.

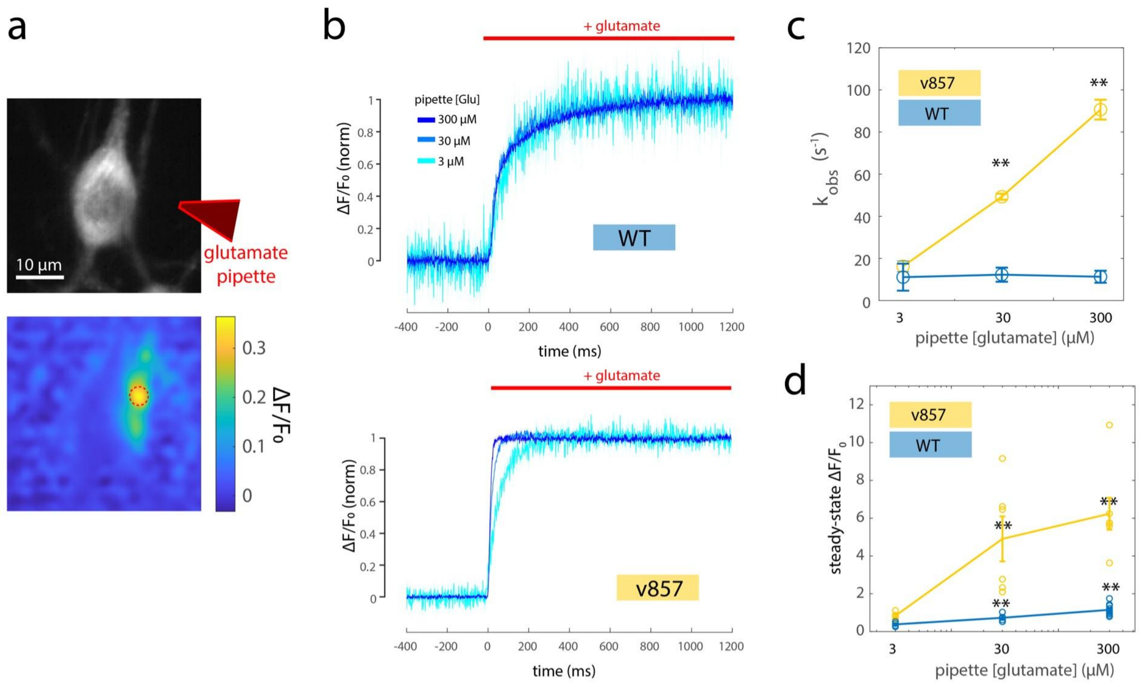

Supplementary Figure 2. On-cell kinetics measured by rapid glutamate application. a) Top, cultured rat hippocampal neurons were approached with a glass pipette containing glutamate buffer, then air pressure was used to puff glutamate onto the cell. Bottom, $\Delta F/F_0$ image for the same cell calculated over a 50 ms time window following flow onset. Red circle denotes the region from which signal was extracted (radius 2 µm centered at response maximum for each cell). b) Mean $\Delta F/F_0$ at 3 pipette glutamate concentrations for WT (top) and iGluSnFR3.v857 (bottom), normalized to steady-state value for each trace. Shaded region denotes SEM. c) ON rates fit using least squares to the mean response for the two indicators at each pipette glutamate concentration. Errorbars denote 95% ci. d) Mean steady-state $\Delta F/F_0$ measured for the two indicators at each pipette glutamate concentration. Errorbars denote SEM. (b-d) N=4,4,10 cells (3,30,300 µM) for WT. N = 5,6,7 cells (3,30,300 µM) for v857. **: p<0.05 vs 3 µM condition, two-sided two-sample t-test.

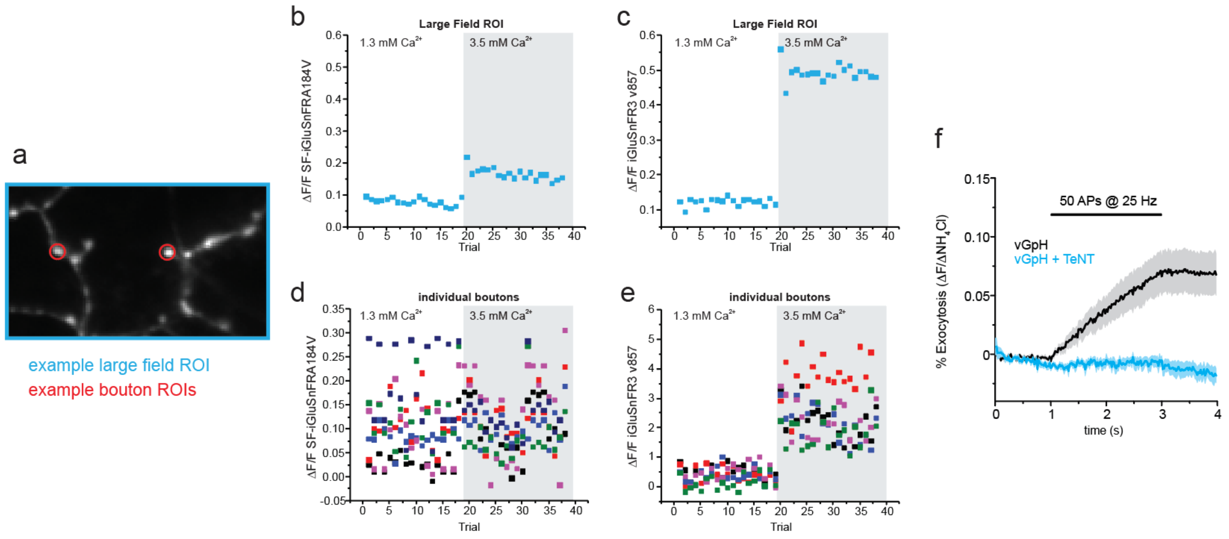

Supplementary Figure 3. Extended data associated with culture field stimulation experiments.. a-e) Associated with Figure 3j,k. Trial-by-trial response amplitudes of iGluSnFR variants over small and large regions of interest. a) Example regions of interest analyzed. ROIs for individual boutons (red) were compared to larger ROIs (blue) containing multiple structures. b-c) Typical example traces for large ROIs, showing an increase in response amplitude following increase of extracellular [Ca^{2+}], for (b) SF-iGluSnFR.A184V and (c) iGluSnFR3.v857. d-e) Typical traces for small ROIs for (d) SF-iGluSnFR.A184V and (e) iGluSnFR3.v857. Responses over all identified boutons under both conditions are plotted in Figure 3k. f) Associated with Figure 3h,i. Effect of TeNT on exocytosis measured using a genetically encoded pH indicator (pHluorin) fused to a vesicular glutamate transporter (vGpH). Control (black; 10 cells) and TeNT-LC (cyan; 14 cells) expressing cells were imaged during field stimulation with 50 APs at 25 Hz. Exocytosis values are normalized to NH_4Cl treatment as previously described (Sankaranarayanan, S., De Angelis, D., Rothman, J.E., and Ryan, T.A. (2000)). Lines denote mean, shaded areas denote SEM.

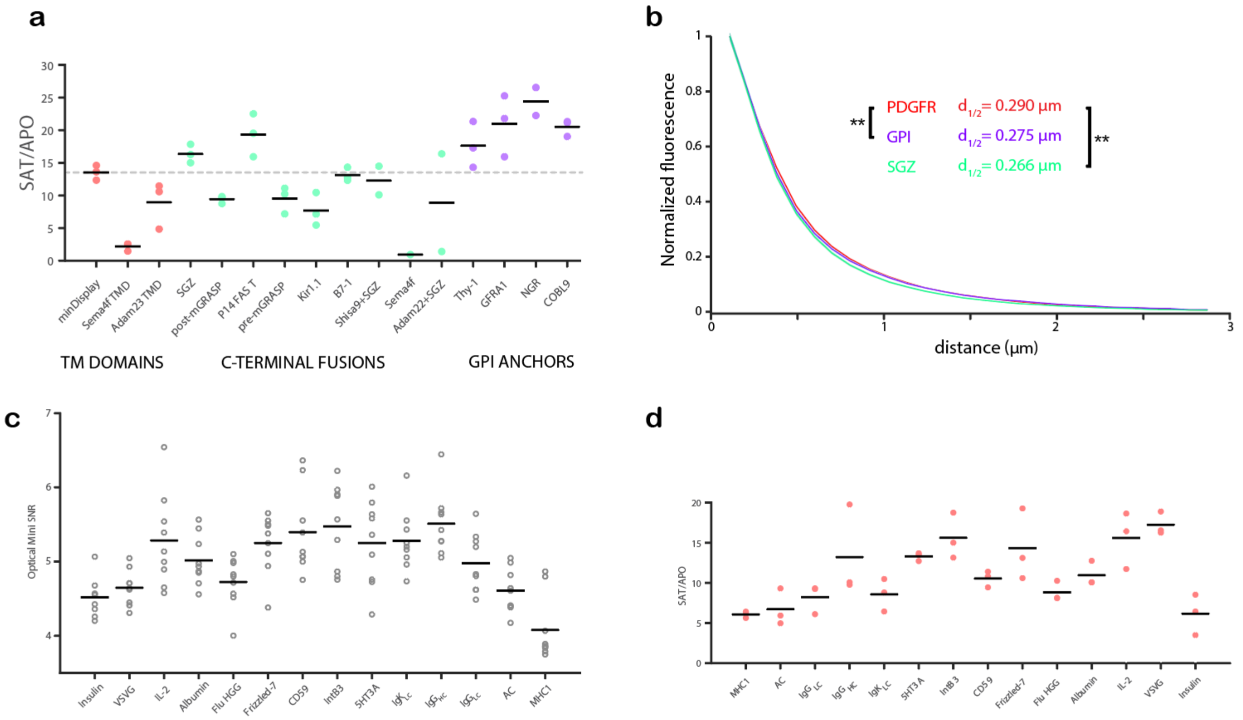

Supplementary Figure 4. Characterization of surface display construct variants. a) Fold brightness change of iGluSnFR3.v857 in different display construct variants upon addition of saturating glutamate (20 mM). n=2-3 biologically independent cultures per construct, as shown. b) Mean optical mini $\Delta F/F_0$, normalized to maximum, as a function of distance from peak for iGluSnFR3.v857 in the PDGFR, GPI, and SGZ backbones, calculated one frame after onset. (PDGFR, $d_{1/2}$ = 0.290+-0.003 μm, n=6593 events; GPI: $d_{1/2}$=0.275+-0.004 μm, n=4421 events, p=0.0014; SGZ, $d_{1/2}$=0.266+-0.003 μm, n=5261 events, p<1e-4; bootstrap test (two-sided)). Shading denotes SEM. c) Mean SNR of optical minis for v857.GPI in the GPI backbone with different N-terminal leader sequences. n=6-9 FOVs per construct, as shown, from 2-3 biologically independent culture wells (3 FOVs per well). d) Fold brightness change of v857.GPI with different N-terminal leader sequences upon addition of saturating glutamate (20 mM). n=2-3 biologically independent culture wells per construct, as shown. In all cases, black lines denote the mean for each group. Sequences for each variant are presented in Supplementary Table 1.

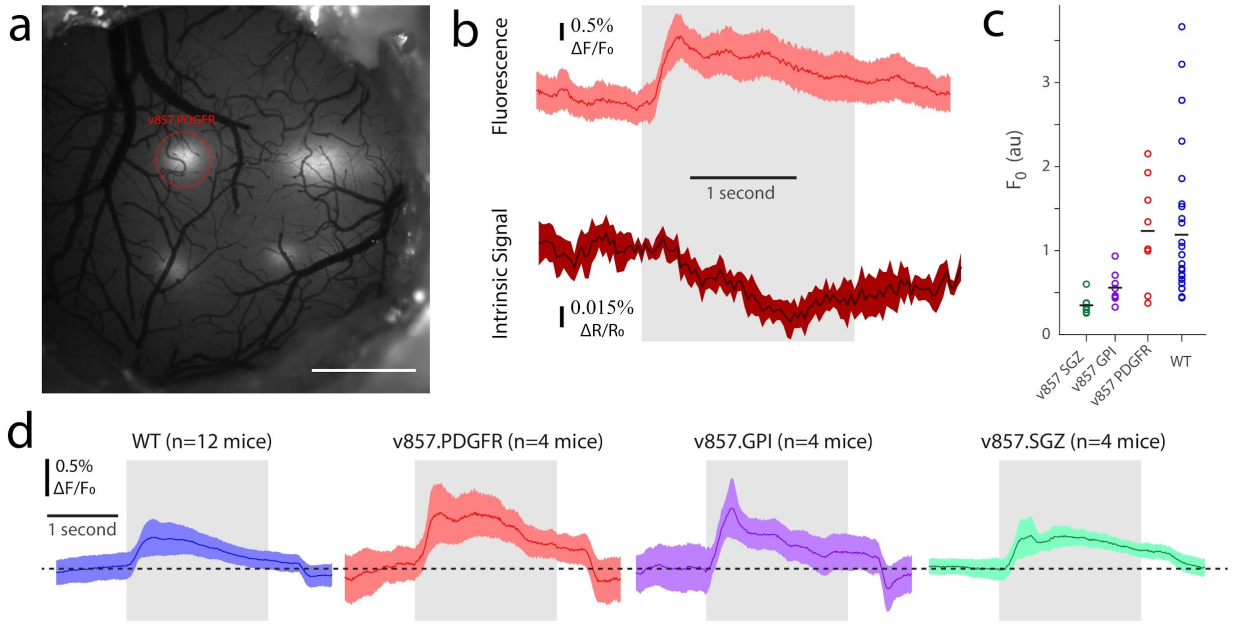

Supplementary Figure 5. 1P cortical imaging of iGluSnFR3 variants. a) Low-magnification image of mouse dorsal cortex with four injection sites. Red circle marks a region injected with v857.PDGFR. Scale bar: 1 mm. b) Mean response (shading denotes SEM) of the region highlighted in (a) to visual motion stimuli over a single session (n=64 stimuli), in fluorescence (top) and intrinsic signal (bottom). Gray region denotes time of visual stimulus. c) Baseline mean brightness of injected regions for 4 iGluSnFR variants, 3 weeks after AAV injection at matched titers. Each marker denotes a field of view (SGZ. n=8 FOVs from 4 mice; GPI n=8 FOVs from 4 mice; PDGFR n=8 FOVs from 4 mice, WT n=32 FOVs from 12 mice). Black lines denote means. d) Mean fluorescence (shading denotes SEM) response across the 50% most responsive sessions across all injection sites, for the 4 constructs. Gray region denotes time of visual stimulus.

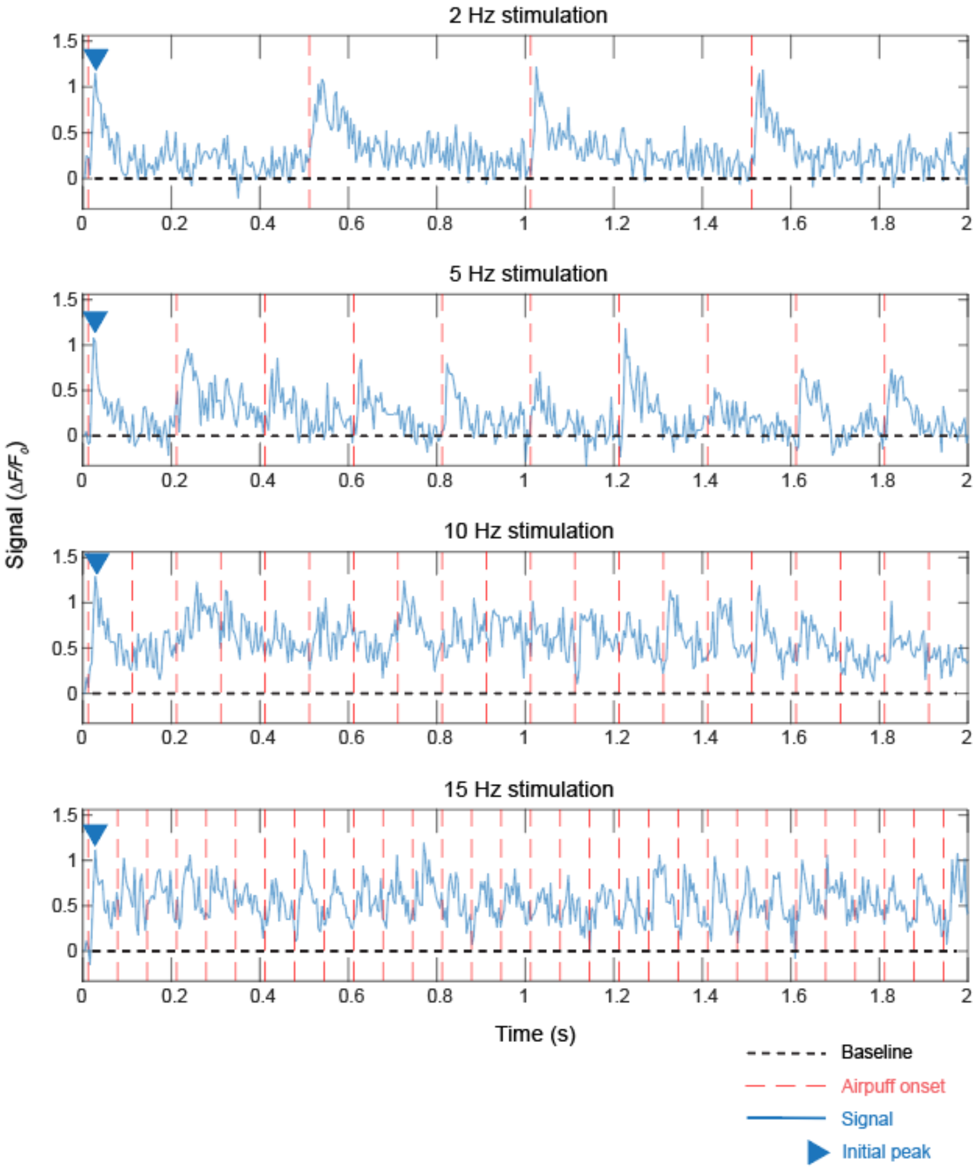

Supplementary Figure 6. First 2 seconds raw trace of an iGluSnFR3.v857.GPI-labeled bouton (ROI #1 in Figure 6d). Signals are shown with stimulation frequency from 2 Hz to 15 Hz.

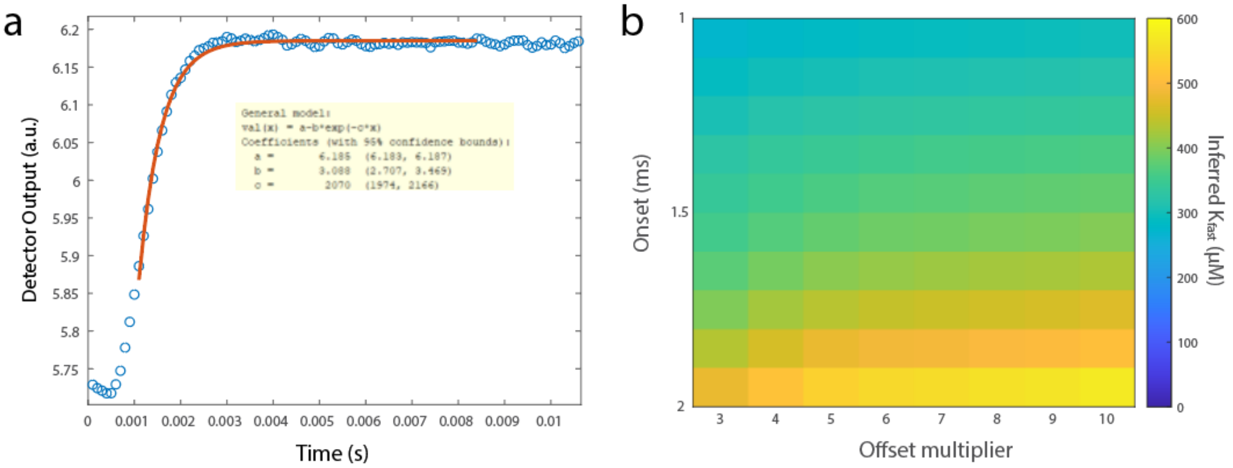

Supplementary Figure 7. Parameter sensitivity of stopped-flow data fitting. a) Example raw data and corresponding fit for a rapid iGluSnFR stopped-flow association reaction in our dataset (SF-iGluSnFR.A184V at 2.5 mM final [glu]). The orange line is the fit exponential saturation function with parameters shown in inset. The dead time of the instrument is visible in the response at times <1 ms. The time-range of the orange curve denotes the time window used for fitting, which is algorithmically selected as described in the Methods. b) To assess sensitivity of inferred kinetic variables to this time window, the two window parameters (onset, in milliseconds, and offset, as a multiple of the 90% rise time t) were systematically varied for the fastest measured indicator, SF-iGluSnFR.A184V. We examined onsets from 1-2 ms and offsets of 3t-10t. Throughout this range, the fit K_{fast} was within a factor of two of the value reported for the parameters we used (1 ms onset, offset 4t). Range of inferred K_{fast} for parameter sweep: 272-573 µM.

Supplementary Tables

Supplementary Table 1. Sequences of surface display construct variants used in the N-terminal screen and C-terminal screen. Sequences I to XXIII show the protein sequences that were used for the N-terminal screen, and constructs XXIV to XL show the amino acid sequences for the C-terminal screen (Figure 4). N-terminal mutations were introduced relative to iGluSnFR3.v857.GPI (sequence XXVI). C-terminal mutations were introduced relative to iGluSnFR.v857 with the IgK N-terminal secretion leader (sequence XIII). All sequences were codon-optimized for *Mus musculus.*

Construct	N-terminal	C-terminal	GenBank ID, Accession ID, or Uniprot #	Reference	Sequence
I	Insulin		AAA59172.1		MALWMRLLPLLALLALWGPDPAAA
II	SPARC		CAG33080.1		MRAWIFFLLCLAGRALA
III	Vesicular Stomatitis Virus G		AAA48403.1		MLSYLIFALAVSPILG
IV	Preprochymotrypsinogen		AAA52128.1		MAFLWLLSCWALLGTTFG
V	Interleukin-2		AAD48509.1		MYRMQLLSCIALSLALVTNSM
VI	Gaussia luciferase		AAG54095.1		MGVKVLFALICIAVAEA
VII	Albumin		AAA98797.1	Kober et al. *Biotechnol. Bioeng.* 110, 1164 (2013)	MKWVTFISLLFLFSSAYS
VIII	Influenza Hemagglutinin		ABB80034.1	T. Patriachi et al. *Science*, 360, eaat4422 (2018)	MKTIIALSYIFCLVLG
IX	Frizzled-7		NP_032083.3		MRGPGTAASHSPLGLCALVLALLGALPTDTRA
X	CD59		CAG46523.1		MGIQGGSVLFGLLLVLAVFCHSGHS
XI	Integrin β pat-3		NP_497787.1	J. Kim et al. *Nat. Methods*, 9, 96 (2012)	MPPSTSLLLLAALLPFALPASDWKTGEVT
XII	5HT3A serotonin receptor			Lorenzen at el. *Sci Adv* 2019 Sep; 5(9): eaaw2778	MRLCIPQVLLALFLSMLTGPGEG
XIII	IgK light chain precursor		AAT37716.1	O. Takenouchi et al. *Sci. Rep*, 8, 677 (2018)	MDMRAPAGIFGFLLVLFPGYRS
XIV	Ig heavy chain			Kober et al. *Biotechnol. Bioeng.* 110, 1164 (2013)	MDWTWRVFCLLAVTPGAHP
XV	Ig light chain		AAA20442.1	Kober et al. *Biotechnol. Bioeng.* 110, 1164 (2013)	MAWSPLFLTLITHCAGSWA
XVI	Azurocidin preproprotein		QIH53806.1	Kober et al. *Biotechnol. Bioeng.* 110, 1164 (2013)	MTRLTVLALLAGLLASSRA
XVII	Cystatin-S precursor		NP_001691.1	Kober et al. *Biotechnol. Bioeng.* 110, 1164 (2013)	MARPLCTLLLLMATLAGALA
XVIII	A conotoxin lp1.3		NP_001890.1	Kober et al. *Biotechnol. Bioeng.* 110, 1164 (2013)	MGMRMMFIMFMLVVLATTVVS
XIX	Aspartic proteinase nepenthesin-1		AAS93426.1	Kober et al. *Biotechnol. Bioeng.* 110, 1164 (2013)	MASSLYSFLLALSIVYIFVAPTHS
XX	Acid chitinase		BAF98915.1	Kober et al. *Biotechnol. Bioeng.* 110, 1164 (2013)	MKTHYSSAILPILTLFVFLSINPSHG
XXI	Cholera toxin		ABF74624.1	Kober et al. *Biotechnol. Bioeng.* 110, 1164 (2013)	MIKLKFGVFFTVLLSSAYA
XXII	MHC class I antigen + prolactin		BAA06291.1	Kober et al. *Biotechnol. Bioeng.* 110, 1164 (2013)	MVPCTLLLLLAAALAPTQTRA SA DSKGSSQKGSRLLLLLVVSNLLLCQGVVSTPV
XXIII	Transferrin receptor		XP_006523736.1 NP_776378.2	B. Chow et al. *Nature*, 463, 98 (2010)	MMDQARSAFSNLFGGEPLSCTRASLARQVDGDNSHVEMKLAVDEEENADNNTKANVTKPKRCSGSICYGTIAVIVFFLIGFMIGYLGY
XXIV		Adam23_TM	KAF6112537.1		KDEGPKGPSATNLIIGSIAGAILVAAIVLGGTGWGFKNVKKRRFDPTQQGPI
XXV		Semaphorin-4F_TM	NP_001295303.1	Jones *et al. Front Genet* 9, 746 (2019)	QRGPANRAHTVVGAGLVGFFLGVLAASLTLLLIGRRQQRRRQRELLARDKVGLDLGAPPSGTTSYSQDPPSPSPEDERLPLALGKRGSGFGGFPPPFLLDSCPSPAHIRLTGAPLATCDETSI
XXVI		GPI_COBL9	NP_199738.1		SDNDYGKDGLAGASGGRRNGAITVLSFITFYVAAFMVLL

XXVII		GPI_NGR	NP_075358.1		AGSGASGTGDAEGSGALPALACSLAPLGLALVLWTVLGPC
XXVIII		GPI_GFRA1	BCP51370.1		SDNDYGKDGLAGASHITTKSMAAPPSCGLSSLPVMVFTALAALLSVSLAETS
XXIX		Adam22_TM_PDZ_TTPV	NM_001324418	Charmsaz *et al. BMC Med 18* (1), 349 (2020)	NAVGQDTQEITLSGNGVAGTNIIIGIIAGTILVLALILGITAWGYKNYREQRQLPDRHKQLRATARATDYLQADAITRIPDYRYRYQRRDRDDDRDTEPDHDRDADPVGVKGFNTLPDTEIDTTPV
XXX		Semaphorin-4F_TM_PDZ_TTPV	NP_001295303	Jones *et al. Front Genet* 9, 746 (2019)	QRGPANRAHTVVGAGLVGFFLGVLAASLTLLLIGRRQQRRRQRELLARDKVGLDLGAPPSGTTSYSQDPPSPSPEDERLPLALGKRGSGFGGFPPPFLLDSCPSPAHIRLTGAPLATCDETSITTPV
XXXI		Shisa9_TM_PDZ_TTPV	A0A6G1B171		NAVGQDTQEVIVPLHDPTKDKTNLIVYIICGVVAVMVLVGIFTKLGLEKAHRPQREDRHKQLRATARATDYLQADAITRIPDYRYRYQRRDRDDDRDTEPDHDRDADPVGVKGFNTLPDTEIDTTPV
XXXII		Nrx1b_TM_PDZ_TTPV	NP_445803.1	Chen *et al. Cell* 170 (6), 1234-1246 (2017)	LQADAITRIPDYRYRYQRRDRDDDRDTEPDHDRDADPVGVKGFNTLPDTEIDTTPV
XXXIII		Thy-1 GPI	NP_033408.1, NM_009382.3		SISVYRDKLVKCGGISLLVQNTSWMLLLLLSLSLLQALDFISL
XXXIV		B7-1	NP_002600	Kaymaz *et al. Turk J Gastroenterol* 32 (12), 1038-1048 (2021)	NAVGQDTQEVIVVPHSLPFKVVVISAILALVVLTIISLIILIKCFCKHRSCFRRNEASRETNNSLTFGPEEALAEQTVFL
XXXV		Kir1.1	NP_033985.3, NM_009855.2	Matundan *et al. J Virol* 95 (19), e0103621 (2021)	NAVGQDTQEVIVVPHSLPFKVVVISAILALVVLTIISLIILIMLWQKKPRYDNPNFVLSEVDETDDTQM
XXXVII		P14 FAST		Corcoran *et al. J Biol Chem* (2006)	KLQVDEQKLISEEDLNAVGQDTQEVIVVPHSLPFKVVVISAILALVVLTIISLIILIKYLQKRRERRRQDRHKQLRATARATDYLQADAITRIPDYRYRYQRRDRDDDRDTEPDHDRDADPVGVKGFNTLPDTEIDTTPV
XXXIX		PDGFR		Marvin *et al.* (2013, 2018); from iGluSnFR and SF-iGluSnFR	AVGQDTQEVIVVPHSLPFKVVVISAILALVVLTIISLIILIMLWQKKPR
XL		SGZ	NP_445803.1 (modified)	Chen *et al. Cell* 170 (6), 1234-1246 (2017)	NAVGQDTQEVIVVPHSLPFKVVVISAILALVVLTIISLIILIMLWQKKPRDRHKQLRATARATDYLQADAITRIPDYRYRYQRRDRDDDRDTEPDHDRDADPVGVKGFNTLPDTEIDNPQTTSV

Supplementary Table 2. Summary of iGluSnFR variants tested in this study. 'WT' refers to SF-Venus-iGluSnFR.A184V.

Variants tested in this study	Reference	Main Figures	Extended Data Figures	Supplementary Figures
SF-iGluSnFR.A184V	*Marvin et al (2018)*	3, 6	9	3, 7
SF-iGluSnFR.A184S	*Marvin et al (2018)*	5		
SF-Venus-iGluSnFR.A184V	*Marvin et al (2018)*	1, 2, 3, 6	Table 1, 1, 2, 3, 4, 5, 7	2, 5
SF-Venus-iGluSnFR.A184S	*Marvin et al (2018)*	1		
iGluSnFR3 v82	*This study*	1, 2, 3, 6	Table 1, 1, 2, 3, 4, 5	
iGluSnFR3 v857	*This study*	1-6	Table 1, 1-6	1, 2, 3, 4, 5, 6, 7
