# Supplementary material for: Glutamate indicators with improved activation kinetics and localization for imaging synaptic transmission
Source: Nat Methods. 2023 May 4;20(6):925–34. doi: 10.1038/s41592-023-01863-6 (PMC10250197; doi:10.1038/s41592-023-01863-6)
Supplement: Supplementary file 2 — Reporting Summary [file 41592_2023_1863_MOESM2_ESM.pdf]

nature research

Corresponding author(s): Kaspar Podgorski

Last updated by author(s): Jan 30 2023

Reporting Summary

Nature Research wishes to improve the reproducibility of the work that we publish. This form provides structure for consistency and transparency in reporting. For further information on Nature Research policies, see our Editorial Policies and the Editorial Policy Checklist.

Statistics

For all statistical analyses, confirm that the following items are present in the figure legend, table legend, main text, or Methods section.

n/a	Confirmed	
☐	☒	The exact sample size (*n*) for each experimental group/condition, given as a discrete number and unit of measurement
☐	☒	A statement on whether measurements were taken from distinct samples or whether the same sample was measured repeatedly
☐	☒	The statistical test(s) used AND whether they are one- or two-sided<br>*Only common tests should be described solely by name; describe more complex techniques in the Methods section.*
☐	☒	A description of all covariates tested
☐	☒	A description of any assumptions or corrections, such as tests of normality and adjustment for multiple comparisons
☐	☒	A full description of the statistical parameters including central tendency (e.g. means) or other basic estimates (e.g. regression coefficient) AND variation (e.g. standard deviation) or associated estimates of uncertainty (e.g. confidence intervals)
☐	☒	For null hypothesis testing, the test statistic (e.g. *F*, *t*, *r*) with confidence intervals, effect sizes, degrees of freedom and *P* value noted<br>*Give P values as exact values whenever suitable.*
☒	☐	For Bayesian analysis, information on the choice of priors and Markov chain Monte Carlo settings
☒	☐	For hierarchical and complex designs, identification of the appropriate level for tests and full reporting of outcomes
☒	☐	Estimates of effect sizes (e.g. Cohen's *d*, Pearson's *r*), indicating how they were calculated

Our web collection on statistics for biologists contains articles on many of the points above.

Software and code

Policy information about availability of computer code

Data collection	The GENIE project team used custom code for data acquisition (lightly modified from Wardill et al 2013; available from the GENIE Project Team upon request). Widefield imaging used WaveSurfer v1.0.6 and Hamamatsu HCImage 4.3.1, and NIS-Elements 4.1. Two photon microscopes were operated using Scanimage (Vidrio Technologies/MBF; versions 2019 through 2021).
Data analysis	Data analysis was performed in Graphpad Prism 8, Matlab R2020a, Fiji (ImageJ) 1.5, and DeepLabCut v2.

For manuscripts utilizing custom algorithms or software that are central to the research but not yet described in published literature, software must be made available to editors and reviewers. We strongly encourage code deposition in a community repository (e.g. GitHub). See the Nature Research guidelines for submitting code & software for further information.

Data

Policy information about availability of data

All manuscripts must include a data availability statement. This statement should provide the following information, where applicable:

- Accession codes, unique identifiers, or web links for publicly available datasets
- A list of figures that have associated raw data
- A description of any restrictions on data availability

Datasets are publicly available on Figshare. doi: 10.25378/janelia.21985406
All figures and supplemental figures have associated raw data.

Field-specific reporting

Please select the one below that is the best fit for your research. If you are not sure, read the appropriate sections before making your selection.

☒ Life sciences ☐ Behavioural & social sciences ☐ Ecological, evolutionary & environmental sciences

For a reference copy of the document with all sections, see nature.com/documents/nr-reporting-summary-flat.pdf

Life sciences study design

All studies must disclose on these points even when the disclosure is negative.

Sample size	In all assays not involving animals, sample sizes were determines using separate pilot datasets, from which the variability of controls was estimated and sample sizes selected that would enable detection of expected effect magnitudes. For in vivo studies, experiments that aimed to quantitatively compare treatment groups used 2-12 animals per group, as described. Other in vivo experiments are demonstrations and involve only a single animal, as described, but are not used for quantitative comparison. Sample sizes for in vivo studies were not selected based upon a power analysis, but were the largest that could feasibly be acquired. The analyses use biological replicates as the unit of variability and the sample sizes are therefore sufficient to support the conclusions drawn.
Data exclusions	Data were excluded from analysis only only the basis of technical failures during data acquisition.
Replication	We are unaware of attempts to replicate these studies precisely. We have shared iGluSnFR3 with many groups (>30) and the performance described to us by these groups is consistent with those reported here.
Randomization	Where possible, individual with similar characteristics were distributed to different treatment groups to control for batch effects, i.e. cage mates were assigned equally to different treatments, and batches of neuron cultures were assigned to both control and treatment conditions. In all other cases, samples were allocated randomly to treatment groups.
Blinding	Investigators were not blinded to group allocation during data collection. Blinding was unnecessary because data collection used highly automated instruments and/or clearly defined protocols that minimize the risk of experimenter bias. The benefit of blinding would not warrant the additional complexity and potential errors associated with it in this context. Similarly, the analyses performed were virtually all automated, making blinding irrelevant. Manual annotation was necessary to identify synapses in expansion microscopy volumes; annotations were not performed blinded, but were subsequently assessed by an expert blinded to treatment group before analysis. Examples shown in figures were either randomly selected or manually selected as representative examples typical of the experiment.

Reporting for specific materials, systems and methods

We require information from authors about some types of materials, experimental systems and methods used in many studies. Here, indicate whether each material, system or method listed is relevant to your study. If you are not sure if a list item applies to your research, read the appropriate section before selecting a response.

Materials & experimental systems

n/a	Involved in the study	
☐	☒	Antibodies
☒	☐	Eukaryotic cell lines
☒	☐	Palaeontology and archaeology
☐	☒	Animals and other organisms
☒	☐	Human research participants
☒	☐	Clinical data
☒	☐	Dual use research of concern

Methods

n/a	Involved in the study	
☒	☐	ChIP-seq
☒	☐	Flow cytometry
☒	☐	MRI-based neuroimaging

Antibodies

Antibodies used	Primary: mouse anti-bassoon #ab82958 (abcam) 1:200, rabbit anti-homer #ab97593 (abcam) 2:200, chicken anti-GFP #ab13970 (abcam) 1:500. Secondary: goat anti-mouse AF594 A-11012 (Invitrogen), goat anti-rabbit Atto 647N 40839-1ML-F (Sigma Aldrich), goat anti-chicken AF 488 A-11039 (Invitrogen).
Validation	These antibodies are widely used for IHC in mice. The formation of closely apposed structures at morphologically defined spines in the present study validates the use of these antibodies for identifying synapses. #ab82958: manufacturer approves for IHC in mice, multiple customer reviews with images showing appropriate localization. #ab97593: manufacturer approves for IHC. Multiple customer reviews with images showing appropriate localization in mouse tissue section. #ab13970: manufacturer approver for IHC across species. Extensive validation on manufacturer website such as images in tranfected and nontransfected tissue.

Animals and other organisms

Policy information about studies involving animals; ARRIVE guidelines recommended for reporting animal research

Laboratory animals	Mice: Emx1-cre (JAX 005628), female, 8-30 wks; C57Bl/6, mixed sexes, 8-30 wks; Scnn1a-Tg3-Cre (JAX 009613), male, 8-30 wks
Wild animals	The study did not involve wild animals.
Field-collected samples	The study did not involve field-collected samples.
Ethics oversight	All experimental procedures involving animals were performed in accordance with protocols approved by the Institutional Animal Care and Use Committee at the respective institute ((HHMI Janelia Research Campus, Dartmouth College, University of California San Diego, and TUM). Procedures in the USA conform to the NIH Guide for the Care and Use of Laboratory Animals. Procedures at TUM were approved by the state government of Bavaria, Germany .<br>HHMI Janelia Research Campus: protocols 14-115, 16-225<br>Dartmouth College: protocol 00002115<br>University of California, San Diego: protocol S02174M<br>Technical University of Munich: ROB-55.2-2532.Vet_02-17-181

Note that full information on the approval of the study protocol must also be provided in the manuscript.
